# A powerful machine learning approach to identify interactions of differentially abundant gut microbial subsets in patients with metastatic and non-metastatic pancreatic cancer

**DOI:** 10.1080/19490976.2024.2375483

**Published:** 2024-07-07

**Authors:** Annacandida Villani, Andrea Fontana, Concetta Panebianco, Carmelapia Ferro, Massimiliano Copetti, Radmila Pavlovic, Denise Drago, Carla Fiorentini, Fulvia Terracciano, Francesca Bazzocchi, Giuseppe Canistro, Federica Pisati, Evaristo Maiello, Tiziana Pia Latiano, Francesco Perri, Valerio Pazienza

**Affiliations:** a Division of Gastroenterology, Fondazione IRCCS Casa Sollievo della Sofferenza, San Giovanni Rotondo, Italy; b Biostatistic Unit, Fondazione IRCCS Casa Sollievo della Sofferenza, San Giovanni Rotondo, Italy; c Proteomics and Metabolomics Facility (ProMeFa), IRCCS San Raffaele Scientific Institute, Milan, Italy; d Scientific Direction, Association for Research on Integrative Oncological Therapies (ARTOI), Roma, Italy; e Abdominal Surgery Unit, IRCCS Casa Sollievo della Sofferenza, San Giovanni Rotondo, Italy; f Histopathology Unit, Cogentech S.C.a.R.L, Milan, Italy; g Oncology Unit, Fondazione IRCCS Casa Sollievo della Sofferenza, San Giovanni Rotondo, Italy

**Keywords:** Microbiota, machine learning, pancreatic cancer, metastasis

## Abstract

Pancreatic cancer has a dismal prognosis, as it is often diagnosed at stage IV of the disease and is characterized by metastatic spread. Gut microbiota and its metabolites have been suggested to influence the metastatic spread by modulating the host immune system or by promoting angiogenesis. To date, the gut microbial profiles of metastatic and non-metastatic patients need to be explored. Taking advantage of the 16S metagenomic sequencing and the PEnalized LOgistic Regression Analysis (PELORA) we identified clusters of bacteria with differential abundances between metastatic and non-metastatic patients. An overall increase in Gram-negative bacteria in metastatic patients compared to non-metastatic ones was identified using this method. Furthermore, to gain more insight into how gut microbes can predict metastases, a machine learning approach (iterative Random Forest) was performed. Iterative Random Forest analysis revealed which microorganisms were characterized by a different level of relative abundance between metastatic and non-metastatic patients and established a functional relationship between the relative abundance and the probability of having metastases. At the species level, the following bacteria were found to have the highest discriminatory power: *Anaerostipes hadrus*, *Coprobacter secundus*, *Clostridium* sp. 619, *Roseburia inulinivorans*, *Porphyromonas* and *Odoribacter* at the genus level, and *Rhodospirillaceae*, *Clostridiaceae* and *Peptococcaceae* at the family level. Finally, these data were intertwined with those from a metabolomics analysis on fecal samples of patients with or without metastasis to better understand the role of gut microbiota in the metastatic process. Artificial intelligence has been applied in different areas of the medical field. Translating its application in the field of gut microbiota analysis may help fully exploit the potential information contained in such a large amount of data aiming to open up new supportive areas of intervention in the management of cancer.

## Introduction

Global statistics updated to 2020 assign pancreatic cancer (PC) in seventh place among the causes of cancer-related death.^1^ Indeed, compared to all other cancers, the 5-year survival rate for PC is among the lowest (10%),^2^ and it further drops to approximately 3% when considering stage IV disease characterized by metastatic spread.^3^ Such poor data are mainly linked to the subtle and insidious onset of PC and its late diagnosis.^4^ In fact, screening methods are not recommended unless in high-risk subjects, in the absence of specific biochemical markers and in the presence of non-typical symptoms in the initial stages of the disease, making early diagnosis difficult to achieve.^5,6^ For these reasons, more than 80% of patients are diagnosed with PC when the disease is locally advanced or metastatic, the tumor is unresectable and palliative chemotherapy is the standard of care.^7–9^ Metastases development is a complex multi-step process that requires cancer cells to detach from the primary tumor, invade the adjacent tissue, migrate into the blood vessels, survive in the bloodstream, leave the circulatory system and colonize a distant body site.^10,11^ Therefore, for productive metastasis establishment, cancer cells must cope with a number of hurdles, and the participation of several actors from the tumor microenvironment (TME) as well as from outside the tumor is required.^12^ A study by Fu *et al*. recently demonstrated that, as a part of the TME, intra-tumor microbiota influence the process of breast cancer metastasis by helping circulating tumor cells re-organize their cytoskeleton to resist mechanical stress in circulation.^13^ However, it has been proposed, that the gut microbiota and its metabolites may be involved at a distance in the process of tumor spread through different mechanisms. One of these is the ability of microbes to modulate the host immune system, thus helping cancer cells to escape immune surveillance^14^ and creating the so-called pre-metastatic niche, a permissive milieu for primary cancer cell engraftment into secondary organ.^15–17^ Another important contribution of gut microbiota to metastasis is the promotion of angiogenesis through stimulation of vascular endothelial growth factor (VEGF) expression by lipopolysaccharide (LPS) and Toll-like receptor-4 (TLR-4).^18,19^

Despite this evidence linking the gut microbiota to the metastatic process, gut bacterial communities characterizing metastatic cancer carriers remain largely uninvestigated.

In the current study, the gut microbial composition of a cohort of patients with either local or metastatic PC was profiled with the aim of identifying bacterial patterns that could discriminate between these two conditions. Moreover, to gain further insight into how the gut microbiota can predict the risk of metastases, a machine learning approach was also performed to determine the interactions of the relative abundance of microorganisms, which may represent bacterial signatures of localized and metastatic PC.

## Materials and methods

### Study participants

Subjects were recruited between June 2019 and April 2022 from the Fondazione IRCCS “Casa Sollievo della Sofferenza” Hospital, San Giovanni Rotondo, Italy, using the same protocols for each patient for collection, processing, and conservation of biological samples. Ethical approval from the IRCCS “Casa Sollievo della Sofferenza” Hospital, under Ethical Committee approval number N.184/CE and written informed consent (IC) were obtained from the study participants. The study was carried out in accordance with the principles of good clinical practice, the Declaration of Helsinki, and in compliance with national legislation. Epidemiological and lifestyle data as well as disease stage, follow-up data, diet, drinking, and smoking habits, were collected at the time of recruitment when the stool specimen was also collected. All data were retrieved from hospital records and collected through direct interviews with the patients. Only patients with PC identified at the time of diagnosis prior to any cancer treatment were included in the study and were classified into two groups according to the presence of metastasis (i.e., non-metastatic and metastatic PC groups). Both patient groups were examined clinically, including assessment of basic anthropometric data and evaluation of blood and serum tests. In addition, patient follow-up data (such as treatment regimen and vital status) were retrieved after the recruitment date for descriptive purposes only and were not utilized in the statistical analyses planned to pursue the purpose of this study, which aimed to assess the presence of differentially abundant bacterial patterns between metastatic and non-metastatic patients, collected at recruitment.

### Laboratory test analysis

All relevant information on blood and serum/plasma analyses was manually extracted from the medical records. Serum glycemia, total protein, transaminases, gamma-glutamyl transferase (GGT), alkaline phosphatase (ALP), creatine kinase (CK), pancreatic amylase, lipase, C-reactive protein (CRP), Carcino-Embryonic Antigen (CEA), Carbohydrate Antigen 19–9 (CA19–9), albumin and bilirubin, as well as erythrocyte sedimentation rate (ESR), blood counts (hemoglobin, white blood cell counts, neutrophils, and platelets) and also prothrombin time (PT) and partial thromboplastin time (PTT) tests were performed according to clinical routine at accredited laboratories at the Fondazione IRCCS “Casa Sollievo della Sofferenza” Hospital, San Giovanni Rotondo, Italy.

### Sample collection and DNA extraction

Each study participant provided a fresh stool sample in a sterile tube which was then stored at −80°C until use. DNA was isolated from a human fecal sample using the QIAamp Fast DNA Stool Mini Kit (Qiagen, Milan, Italy, Cat. N° 51604) according to the manufacturer’s instructions. To optimize the ratio of non-human to human DNA, cells that were difficult to dissolve (such as Gram-positive bacteria) were lysed by heating the fecal suspension at 90°C for 5 min. DNA was checked for concentration and purity at the end of the isolation protocol and stored at −30°C until use.

### Next-generation sequencing and analysis of bacterial 16S rRNA gene

For each sample, 12.5 ng of DNA obtained through the above procedure was used to amplify the V3-V4 region of the 16S rRNA gene using KAPA HiFi HotStart Ready Mix (Roche Diagnostics, Milan, Italy, Cat. N° 07958935001) and the following primers selected by Klindworth^20^ with Illumina adapters added: forward primer: 5'-TCGTCGGCAGCGTCAGATGTGTATAAGAGACAGCCTACGGGNGGCWGCAG reverse primer: 5’-GTCTCGTGGGCTCGGAGATGTGTATAAGAGACAGGACTACHVGGGTATCTAATCC. Samples were then barcoded with the Nextera XT Index Kit (Illumina, Milan, Italy, Cat. N° FC-131-1002), as previously described.^21^ Next, the libraries were purified, quantified using a Qubit™ Flex Fluorometer (Thermo Scientific, Milan, Italy), pooled and sequenced in pairs (2 × 300 cycles) on an Illumina MiSeq platform (San Diego, CA, USA). FASTQ files generated by MiSeq were deposited in ArrayExpress under the code E-MTAB-12513 and de-multiplexed and analyzed using the 16S Metagenomics GAIA 2.0 software, Sequentia Biotech, Barcelona, Spain (2019). Read pairs were quality-controlled (i.e., trimming, clipping and adapter removal) based on FastQC and BBDuk and, to obtain the taxonomic profile of each sample, they were mapped with BWA-MEM against the 16S reference database available in NCBI GenBank. Specifically, all full-length sequences of the 16S rRNA gene from all prokaryotes are included except those longer than 3000 bp which are filtered out, since the gene is expected to be shorter. A prediction of the microbial functions was carried out using the Tax4Fun2 package in R. The Mann–Whitney test was used to analyze the differences of the Kyoto Encyclopedia of Genes and Genomes (KEGG) metabolic pathways between the two groups, which were then depicted using STAMP software.

### Metabolomics analysis

The metabolomics and lipidomics analyses of feces from a subgroup of 20 metastatic and non-metastatic patients were performed using ultra high pressure liquid chromatography (UPLC 1290 system, Agilent Technologies) directly connected to mass spectrometry (TripleTOF 5600+ mass spectrometer, SCIEX equipped with an electrospray ionization source (ESI)). Detailed protocol is reported in Supplementary Material 3.

### Statistical methods

Patient characteristics were reported as medians along with interquartile ranges (i.e., first-third quartiles) and observed and relative frequencies for continuous and categorical variables, respectively. For each continuous variable, the assumption of normality distribution was assessed using the Shapiro–Wilks test, and if the condition was met, mean ± standard deviation (SD) was reported instead of median. Comparisons between patients with and without metastases at enrollment were performed using a two-sample *t*-test (or Mann–Whitney *U* test as appropriate) and chi-square test with Yates’ continuity correction (or Fisher exact test as appropriate) for continuous and categorical variables, respectively. Patients follow-up was defined as the time elapsed between the date of enrollment and death or the date of the last visit, whichever occurred first. The annual mortality rate was reported as the number of death events per 100 person-years and a Poisson regression model was used to assess the differences between the two groups. Stacked bar charts were used to show the gut microbiota composition (i.e., mean relative abundance %) at the phylum, family, genus, and species levels between patient groups. To identify clusters of bacterial populations such that the linear combination of their abundances is differential between patients with and without metastases, the PEnalized LOgistic Regression Analysis (PELORA)^22^ was performed on logit-transformed and standardized relative abundance (i.e. Z-score), as already shown elsewhere.^23–25^ When the relative abundance was exactly 0%, to compute a Z-score this quantity was replaced by 0.0001% (i.e. half of the lowest relative abundance found in the entire dataset). The mean Z-scores of all the bacteria included in a cluster were defined as “the centroid of the cluster”. Multiple clusters of bacterial populations at different taxonomic levels were identified using this algorithm. However, to ease the interpretation of the results, a maximum of two informative clusters were considered for each scenario. Each identified cluster included bacterial populations whose relative abundances were consistently higher (or lower) in metastatic patients than in non-metastatic patients. The optimal penalty parameter value was chosen as the one that achieved the lowest median misclassification rate, computed after several data resamplings (bootstrapping). Box plots and scatterplots of the Z-scores computed at cluster centroids and heatmaps of the relative bacteria abundance (%) identified by PELORA within each cluster are shown at different taxonomic hierarchies. As stated by Dettling and Buhlmann,^22^ the PELORA algorithm “aims to identify multiple class-separating groups (i.e., clusters) such that each group exhibits a good trade-off between expected differential expression and conditional variance of the group mean, and such that the groups together contribute most in predicting the response”. In short, to form a new group of bacteria, their strategy proceeds in a ‘cautious’ manner, starting from scratch and relying on the incremental growth of the group by adding one bacterium after another until the L2 penalized negative log-likelihood function value, defined as the sum of the negative binomial log-likelihood (i.e. likelihood of the data) and the magnitude of the coefficients (L2 “ridge” penalty), in the group stabilizes and cannot be further minimized at the chosen penalty parameter. Once a group was found to be terminated, a new group was started, and the composition of the former groups remained unchanged, while they could still have an effect on the construction of the new group. Please refer to section 3.4.2 of the cited article^22^ for the detailed steps concerning the implementation of the PELORA algorithm.

Furthermore, to delve deeper into the data, the iterative Random Forest (iRF) algorithm^26^ was performed on the same structured dataset as the PELORA analysis (i.e. one record per patient and a set of variables in which the presence of metastasis, clinical information and the relative abundance of each bacterium with respect to each taxonomic classification, respectively, was recorded). It should be noted that the analysis performed with iRF was independent of that performed with the PELORA algorithm. The iRF is a generalization of the Random Forest (RF) algorithm and is commonly used to train a set of feature-weighted decision trees to detect stable and high-order interactions.^26^ In the iRF algorithm, a RF is run iteratively from one to 100 times and, at the last iteration, the feature-weighted RF is ‘mapped’ extracting and applying its decision rules to convert continuous or categorical features into binary variables, a crucial step as it allows the identification of all prevalent interactions. The proportion of times (out of several bootstrap samples) an interaction appears defines a “stability score” (i.e., 0 = totally unstable interaction, 1 = totally stable interaction). To enable iRF training, a “tuning phase” was performed to set its internal parameters. A similar application of the method has been extensively reported in,^27^ and additional information about iRF algorithms along with its “tuning phase” can be found in the Supplemental Statistical Methods. Accumulated Local Effects (ALE) were performed to depict the functional relationship between the relative abundances of the most important bacteria and the probability of having metastases whereas Partial Dependence Plots (PDP) were performed to investigate the “most stable” interactions between two important bacteria, locating all combinations of relative abundance at high risk of metastasis. The discriminatory ability of the iRFs was assessed by the Area Under the ROC Curve (AUC) on the out-of-bag data, along with its 95% CI computed after 1000 stratified bootstrap replicates. Statistical significance was set at *p* < 0.05. All statistical analyses and plots were performed using R (R Development Core Team 2008, version 4.2, packages: supclust, iRF, iml, ggplot2, ggpubr, ComplexHeatmap, pROC, igraph, ggraph). Concerning the association analysis, pairwise Spearman correlations between the gut bacteria discriminating the two cohorts of patients according to AI and the metabolomics compounds were performed, and the Spearman’s rank correlation coefficients (r) were calculated. Results were considered significant when *p* < 0.05.

## Results

### Sample characteristics

Fifty-three patients with pancreatic tumors were enrolled in this study, including 25 with non-metastatic tumors (mean age at enrollment: 65.7 years) and 28 with metastatic tumors (mean age at enrollment: 68.7 years). For brevity, the acronyms “PC” and “PC met” were used to refer to these two groups. Anthropometric, demographic and clinical data are presented in Table 1. The two groups were balanced with the respect to all examined characteristics except for the variables such as: a) characteristics of the tumor, including tumor stage (*p* < .001) which was more advanced in metastatic patients (85.7% of PC met versus 0% of the PC was at stage IV), jaundice (*p* = .004) and endoprosthesis positioning (*p* = .010) which were both more abundant in PC versus PC met patients; b) treatment regimen (after stool collection) like therapeutic plan (*p* = .002), neo-adjuvant chemotherapy (*p* < .001) and radiotherapy (*p* = .001) which were mainly administered to PC group whereas PC met mainly underwent, first-line chemotherapy (*p* = .016) and radiotherapy (*p* = .001); c) biochemical tests at the enrollment visit, notably hemoglobin (*p* = .040), red blood cells (*p* = 0.022) and albumin (*p* = 0.041) which were lower in PC than in PC met patients, bilirubin (*p* = .014), aspartate aminotransferase (*p* = .033), GGT (*p* = .046) and ALP (*p* = .038) which were higher in PC than in PC met patients and d) mortality rate, namely, follow-up time from enrollment which was shorter in PC met (*p* = .003) and annual mortality rate which was higher in PC met (*p* = .002).

**Table 1.** Characteristics of patients with pancreatic tumor with and without metastases at the enrollment.

Variable	Category	All subjects (*N* = 53)	PC (*N* = 25)	PC met (*N* = 28)	*p*-value
*Clinical characteristics*	—	—	—	—	—
Age at enrollment (years)	Mean±SD	67.3 ± 10.3	65.7 ± 11.2	68.7 ± 9.4	.290*
Gender – N(%)	Males	33 (62.3)	15 (60.0)	18 (64.3)	.970^§^
BMI at enrollment (Kg/m^2^)	Median [IQR]	24.7 [22.9–27.9]	24.0 [23.0–27.6]	25.2 [22.8–28.0]	.936^
Comorbidities – N(%)	Yes	42 (79.2)	20 (80.0)	22 (78.6)	1.000^§^
	Hypertension	31/42 (73.8)	16/20 (80.0)	15/22 (68.2)	.604^§^
	Diabetes	21/42 (50.0)	13/20 (65.0)	8/22 (36.4)	.122^§^
	Heart disease	13/42 (31.0)	5/20 (25.0)	8/22 (36.4)	.644^§^
	Hypercholesterolemia	8/42 (19.0)	6/20 (30.0)	2/22 (9.1)	.123^#^
	Others	16/42 (38.1)	6/20 (30.0)	10/22 (45.5)	.476^§^
*Tumor characteristics*	—	—	—	—	—
Tumor localization – N(%)	Body	14 (26.4)	3 (12.0)	11 (39.3)	.054^#^
	Head	34 (64.2)	20 (80.0)	14 (50.0)	
	Tail	5 (9.4)	2 (8.0)	3 (10.7)	
Tumor stage – N(%)	IB	2 (3.8)	1 (4.0)	1 (3.6)	<.001^#^
	IIA	1 (1.9)	1 (4.0)	0 (0.0)	
	IIB	1 (1.9)	1 (4.0)	0 (0.0)	
	III	1 (1.9)	0 (0.0)	1 (3.6)	
	IV	24 (45.3)	0 (0.0)	24 (85.7)	
	Unknown (not reported)	24 (45.3)	22 (88.0)	2 (7.1)	
Surgical procedure – N(%)	None	45 (84.9)	21 (84.0)	24 (85.7)	.344^#^
	Duodenocephalopancreasectomy	4 (7.5)	2 (8.0)	2 (7.1)	
	Splenopancreatectomy	2 (3.8)	2 (8.0)	0 (0.0)	
	Liver metastasectomy or atypical resection	2 (3.8)	0 (0.0)	2 (7.1)	
Site of metastases – N(%)	Liver	NA	NA	20 (71.4)	NA
	Lymph nodes	NA	NA	9 (32.1)	
	Peritoneum	NA	NA	9 (32.1)	
	Other sites	NA	NA	5 (17.9)	
Other tumors – N(%)	No	39 (73.6)	18 (72.0)	21 (75.0)	.266^#^
	Bladder or prostatic cancer	4 (7.5)	2 (8.0)	2 (7.1)	
	Breast cancer	2 (3.8)	2 (8.0)	0 (0.0)	
	Ovarian cancer	2 (3.8)	2 (8.0)	0 (0.0)	
	Other (unspecified)	4 (7.5)	1 (4.0)	3 (10.7)	
	Unknown/Not reported	2 (3.8)	0 (0.0)	2 (7.1)	
Family history of cancer – N(%)	Yes	19 (35.8)	8 (32.0)	11 (39.3)	.791^§^
Jaundice- N(%)	Yes	22 (41.5)	16 (64.0)	6 (21.4)	.004^§^
Endoprosthesis – N(%)	Yes	21 (39.6)	15 (60.0)	6 (21.4)	.010^§^
*Concomitant drugs* *(taken during the enrollment visit)*	—	—	—	—	—
Antihypertensive anticoagulant cardioprotective drugs – N(%)	Yes	25 (47.2)	14 (56.0)	11 (39.3)	.112^§^
Antidiabetic drugs pancreatic enzymes – N(%)	Yes	14 (26.4)	7 (28.0)	7 (25.0)	.144^§^
Antibiotics – N(%)	Yes	3 (5.7)	1 (4.0)	2 (7.1)	.104^#^
Statins – N(%)	Yes	4 (7.5)	1 (4.0)	3 (10.7)	.122^#^
Other drugs – N(%)	Yes	12 (22.6)	5 (20.0)	7 (25.0)	.146^#^
*Lifestyle info*	—	—	—	—	—
Smoking status – N(%)	No	29 (54.7)	14 (56.0)	15 (53.6)	.412^#^
	Former smoker	8 (15.1)	2 (8.0)	6 (21.4)	
	Current smoker	16 (30.2)	9 (36.0)	7 (25.0)	
Diet – N(%)	Diabetic diet (or hypoglucide) diet	12 (22.6)	7 (28.0)	5 (17.9)	.158^#^
	Hyperproteic diet	3 (5.7)	3 (12.0)	0 (0.0)	
	Mediterranean and hyperglucide diet	2 (3.8)	1 (4.0)	1 (3.6)	
	Mediterranean diet w/wo more vegetables	36 (67.9)	14 (56.0)	22 (78.6)	
*Treatment regimen (after the stool sampling date)*	—	—	—	—	—
Therapeutical plan – N(%)	No therapy	20 (37.7)	8 (32.0)	12 (42.9)	.002^#^
	RT only	1 (1.9)	0 (0.0)	1 (3.6)	
	Neoadjuvant CT only	5 (9.4)	4 (16.0)	1 (3.6)	
	First-line CT only	10 (18.9)	1 (4.0)	9 (32.1)	
	Adjuvant CT only	1 (1.9)	1 (4.0)	0 (0.0)	
	Neoadjuvant and first-line CT	1 (1.9)	1 (4.0)	0 (0.0)	
	Neoadjuvant and first-line CT+RT	2 (3.8)	2 (8.0)	0 (0.0)	
	Neoadjuvant CT+RT	5 (9.4)	5 (20.0)	0 (0.0)	
	First-line and second-line CT	5 (9.4)	1 (4.0)	4 (14.3)	
	First-line CT+RT	2 (3.8)	1 (4.0)	1 (3.6)	
	Adjuvant and first-line CT+RT	1 (1.9)	1 (4.0)	0 (0.0)	
Neoadjuvant CT – N(%)	No	40 (75.5)	13 (52.0)	27 (96.4)	<.001^#^
	Folfirinox	7 (13.2)	7 (28.0)	0 (0.0)	
	Folfox	1 (1.9)	1 (4.0)	0 (0.0)	
	Gemcitabine	5 (9.4)	4 (16.0)	1 (3.6)	
Neoadjuvant CT: treatment response – N(%)	None	3 (23.1)	2 (16.7)	1 (100.0)	.615^#^
	Unknown (ongoing)	2 (15.4)	2 (16.7)	0 (0.0)	
	Stable disease	5 (38.5)	5 (41.7)	0 (0.0)	
	Stable disease/partial response	2 (15.4)	2 (16.7)	0 (0.0)	
	Disease progression	1 (7.7)	1 (8.3)	0 (0.0)	
First-line CT – N(%)	No	32 (60.4)	18 (72.0)	14 (50.0)	.016^#^
	Folfirinox	3 (5.7)	0 (0.0)	3 (10.7)	
	Gemcitabine	4 (7.5)	2 (8.0)	2 (7.1)	
	Gemcitabine+ Nab-Paclitaxel	11 (20.8)	2 (8.0)	9 (32.1)	
	Other	3 (5.7)	3 (12.0)	0 (0.0)	
First-line CT: treatment response – N(%)	None	11 (52.4)	4 (57.1)	7 (50.0)	.932^#^
	Unknown (ongoing)	3 (14.3)	1 (14.3)	2 (14.3)	
	Stable disease	4 (19.0)	2 (28.6)	2 (14.3)	
	Partial remission/partial response	2 (9.5)	0 (0.0)	2 (14.3)	
	Partial remission	1 (4.8)	0 (0.0)	1 (7.1)	
Second-line CT – N(%)	No	48 (90.6)	24 (96.0)	24 (85.7)	.613^#^
	Folfiri	4 (7.5)	1 (4.0)	3 (10.7)	
	Gemcitabine+ Nab-Paclitaxel	1 (1.9)	0 (0.0)	1 (3.6)	
Second-line CT: treatment response – N(%)	None	4 (80.0)	1 (100.0)	3 (75.0)	1.000^#^
	Unknown (ongoing)	1 (20.0)	0 (0.0)	1 (25.0)	
RT – N(%)	Yes	11 (26.8)	9 (56.2)	2 (8.0)	.001^#^
RT: treatment response – N(%)	None	5 (62.5)	4 (66.7)	1 (50.0)	1.000^#^
	Unknown (ongoing)	2 (25.0)	1 (16.7)	1 (50.0)	
	Stable disease	1 (12.5)	1 (16.7)	0 (0.0)	
*Biochemical exams at enrollment visit*	—	—	—	—	—
Hemoglobin (g/dL)	Median [IQR]	12.8 [11.1–14.0]	11.6 [10.2–12.9]	13.2 [11.9–14.1]	.040^
RBC (M/µL)	Median [IQR]	4.4 [3.9–4.7]	4.1 [3.4–4.5]	4.5 [4.3–4.8]	.022^
PLT (K/µL)	Median [IQR]	259.0 [189.0–300.0]	213.0 [188.0–297.0]	278.5 [219.8–310.8]	.159^
WBC (K/µL)	Median [IQR]	7.3 [6.0–9.3]	6.9 [5.3–8.9]	7.5 [6.1–9.5]	.165^
Neutrophils (K/µL)	Median [IQR]	4.8 [3.6–6.5]	4.5 [3.5–6.0]	5.1 [3.8–6.7]	.402^
Eosinophils (K/µL)	Median [IQR]	0.1 [0.1–0.2]	0.1 [0.1–0.2]	0.1 [0.1–0.2]	.464^
Basophils (K/µL)	Median [IQR]	0.0 [0.0–0.0]	0.0 [0.0–0.1]	0.0 [0.0–0.0]	.442^
Lymphocytes (K/µL)	Median [IQR]	1.5 [1.1–2.0]	1.5 [1.1–1.9]	1.6 [1.1–2.1]	.470^
Monocytes (K/µL)	Median [IQR]	0.4 [0.4–0.6]	0.4 [0.3–0.5]	0.4 [0.4–0.6]	.092^
Glycemia (mg/dL)	Median [IQR]	108.0 [89.0–163.5]	110.0 [88.0–158.5]	107.5 [92.0–164.5]	.992^
Total Protein (g/dL)	Median [IQR]	6.6 [6.3–7.1]	6.4 [5.8–6.9]	6.8 [6.4–7.2]	.158^
Albumin (g/dL)	Median [IQR]	3.4 [2.8–3.7]	3.2 [2.7–3.5]	3.5 [3.3–3.9]	.041^
Bilirubin (mg/dL)	Median [IQR]	0.9 [0.5–8.8]	5.6 [0.8–10.0]	0.6 [0.4–1.5]	.014^
AST (IU/L)	Median [IQR]	36.0 [17.0–100.0]	73.0 [22.0–147.0]	25.5 [16.0–65.8]	.033^
ALT (IU/L)	Median [IQR]	63.0 [24.0–163.0]	122.0 [25.0–288.0]	38.0 [23.0–77.5]	.072^
GGT (IU/L)	Median [IQR]	224.0 [37.0–642.0]	554.0 [44.0–1118.0]	135.5 [27.8–331.5]	.046^
ALP (IU/L)	Median [IQR]	256.0 [90.0–440.0]	366.0 [161.0–565.0]	155.0 [84.0–336.0]	.038^
aPTT (SEC)	Median [IQR]	24.5 [23.0–25.6]	24.8 [24.2–25.7]	24.0 [22.4–25.5]	.084^
aPTT (Ratio)	Median [IQR]	0.9 [0.9–1.0]	0.9 [0.9–1.0]	0.9 [0.9–1.0]	.092^
PT (INR)	Median [IQR]	1.1 [1.0–1.1]	1.1 [1.0–1.1]	1.1 [1.0–1.1]	.721^
Pancreatic amylase (IU/L)	Median [IQR]	27.0 [17.5–38.0]	30.5 [21.0–57.0]	19.0 [16.5–33.5]	.133^
Lipase (U/L)	Median [IQR]	190.0 [87.0 –440.0]	417.5 [94.5–829.2]	134.0 [98.0–205.0]	.069^
Creatin kinase (U/L)	Median [IQR]	60.0 [39.5–88.8]	59.5 [37.8–100.2]	63.0 [46.5–77.5]	.811^
Creatinin (mg/dL)	Median [IQR]	0.7 [0.6–1.0]	0.7 [0.6–1.0]	0.8 [0.7–1.0]	.214^
CEA (ng/mL)	Median [IQR]	2.9 [1.2–8.6]	2.4 [1.1–5.4]	3.3 [1.3–13.4]	.299^
CA 19–9 (U/mL)	Median [IQR]	319.7 [44.2– 3889.4]	223.7 [78.0–2551.5]	659.8 [36.7–6602.9]	.305^
ESR (mm)	Median [IQR]	53.0 [29.0–77.5]	59.0 [30.5–73.5]	45.5 [27.5–79.5]	.570^
CRP (mg/dL)	Median [IQR]	1.5 [0.6–3.2]	1.5 [0.7–2.8]	1.4 [0.6–3.4]	.861^
*Mortality rate*	—	—	—	—	—
Follow-up time (months) from enrollment	Median [IQR]	7.7 [3.0–11.7]	10.1 [7.2–13.1]	4.4 [1.4–9.6]	.003^
Vital status – N(%)	Dead	33 (62.3)	12 (48.0)	21 (75.0)	.053^#^
Annual mortality rate (events/PYs)	N. death events/PYs	33/41	12/26	21/15	.002°
	Rate (n.events per 100PYs)	79.6	45.6	138.7	

*p-value from the two-sample test.

^†^*p*-value from Mann-Whitney U test.

^‡^*p*-value from chi-square test with Yates’ continuity correction.

^§^*p*-value from Fisher’s exact test.

°p-value from the Poisson model.

**Abbreviations:** PC: patients with pancreatic tumor without metastasis at enrollment; PC met: patients with pancreatic tumor with metastasis at enrollment; RT: Radiotherapy; CT: Chemotherapy; SD: Standard deviation; IQR: Interquartile Range (i.e., first-third quartiles); NA: not available; PYs: Person-Year.

### Comparison of fecal microbiota composition between PC patients with or without metastases

**Figure 1.**
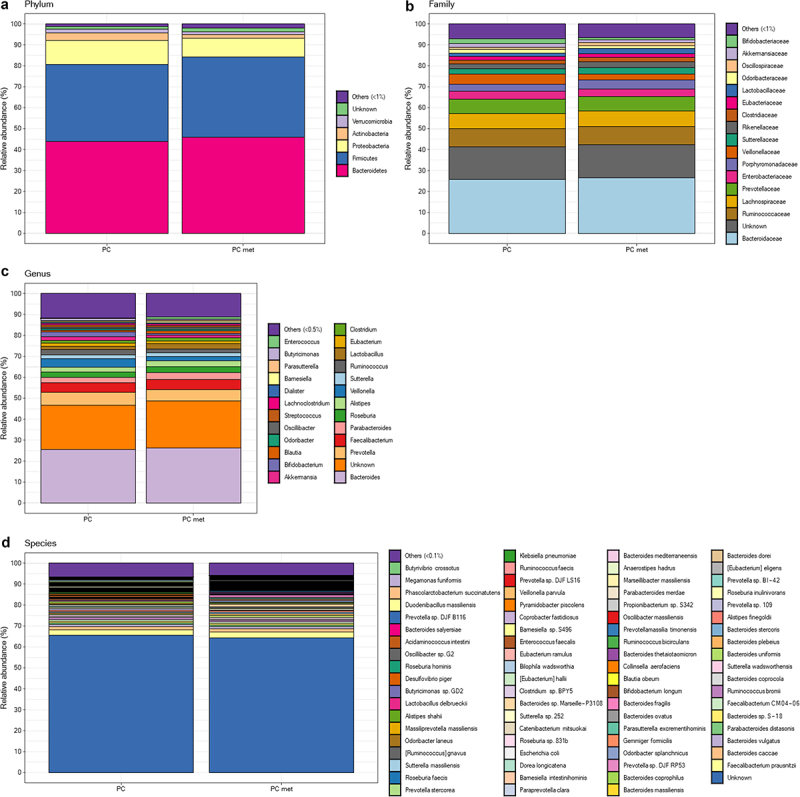
Gut microbiota composition at different taxonomic levels grouped by patients with or without metastases.

**Figure 2.**
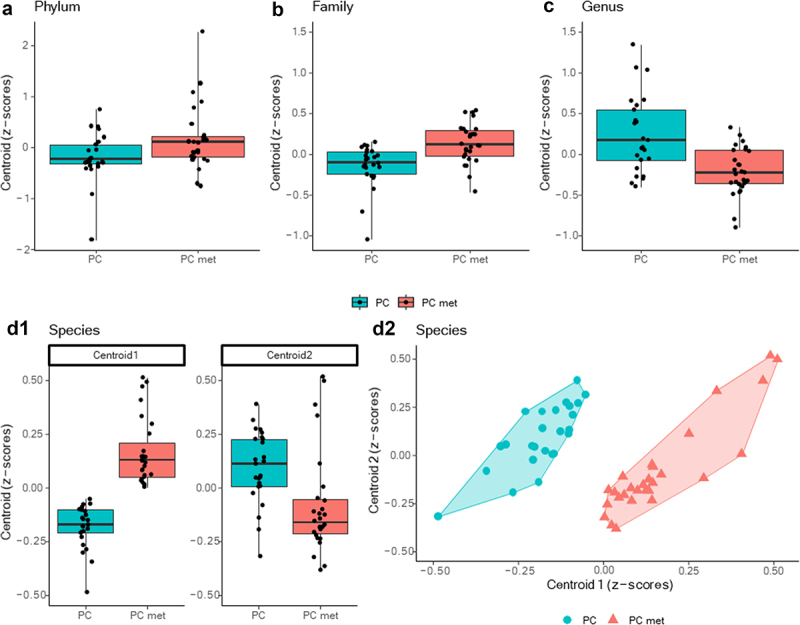
Box plots of the Z-scores computed by PELORA at different taxonomic levels.

**Figure 3.**
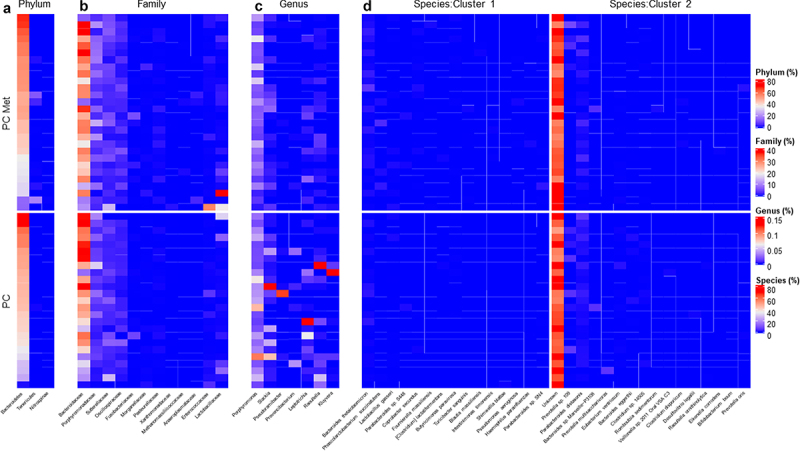
Heatmaps of relative abundance of bacterial populations identified by the PELORA within each cluster.

To highlight any differences in the composition of the intestinal microbiota in the PC and PC met groups, 16S rRNA gene sequencing was performed, producing an average of 183,749.283 (±129203.7919) read pairs for each of the 53 study participants. Alpha-diversity indices (Shannon and Chao1) were calculated both at the genus and the species level in order to analyze the within-sample diversity of the bacterial profiles, but no significant difference emerged between the two groups (data not shown). Figure 1 shows the gut microbiota composition at the phylum (A), family (B), genus (C) and species (D) levels, expressed as relative abundance (%) in the PC and PC met groups. Taxonomic analysis data were used to carry out the PELORA algorithm to detect differences in the microbial population in the two groups of patients and identify specific bacterial patterns. All identified clusters are reported in Table 2. The phyla *Tenericutes*, *Bacteroidetes* and *Nitrospinae* were found to be part of a bacterial cluster and were all enriched in the PC met group compared to the PC group. Another cluster was identified at the family level, including the families of *Anaeroplasmataceae*, *Sutterellaceae*, *Methanomassillicoccaceae, Pasteurellaceae, Porphyromonadaceae, Lactobacillaceae, Oscillospiraceae, Bacteroidaceae, Enterococcaceae, Fusobacteriaceae, Morganellaceae* and *Xanthomonadaceae*, which were all increased in PC met compared to PC (mean Z-scores of cluster centroid: −0.149 *vs* 0.133, *p* < .001). When comparing the two groups at the genus level, the linear combination of abundances revealed another cluster including *Provencibacterium*, *Porphyromonas*, *Raoultella*, *Pseudoramibacter*, *Kluyvera*, *Slackia* and *Leptotrichia*, all of which decreased in PC met with respect to PC (mean Z-scores of cluster centroids: 0.241 vs. −0.216, *p* < .001). At the species level, the PELORA algorithm identifies two clusters. The first one included *Coprobacter secundus*, *Turicibacter sanguinis*, *Phascolarctobacterium succinatutens*, *Bacteroides thetaiotaomicron, Pseudomonas aeruginosa*, *Intestinimonas timonensis*, *Lactobacillus gasseri*, *Blautia massiliensis*, *Parabacteroides* sp. S448, *Haemophilus parainfluenzae*, *Fournierella massiliensis*, *Parabacteroides* sp. SN4, *Butyricimonas paravirosa*, *Shimwellia blattae* and *Clostridium lactatifermentans*, which were all increased in PC met versus PC, with the exception of *S. blattae* (mean Z-scores of cluster centroids: −0.181 *vs* 0.162, *p* < .001). The second species cluster included *Eubacterium ventriosum*, *Raoultella ornithinolytica, Bacteroides* sp. Marseille-P3108, *Clostridium disporicum*, *Veillonella* sp. 2011 Oral VSA C3, *Bacteroides eggerthii*, *Prevotella oris*, *Prevotella* sp. 109, *Bifidobacterium boum*, *Romboutsia sedimentorum*, *Prevotella multisaccharivorax*, *Desulfovibrio legallii*, *Eikenella corrodens*, *Clostridium* sp. 14505, and unknown bacteria, which were decreased in PC met compared to PC patients, with the exception of *C. disporicum* and *P. oris* (mean Z-scores of cluster centroids: 0.093 *vs* −0.083, *p* = 0.003). At this stage of the analysis, it is important to emphasize that two sample t-tests were performed for the mere purpose of providing a general descriptive comparison of cluster centroids and not for drawing inferential conclusions. For each taxonomic level, Figure 2 shows the distribution of Z-scores computed at the cluster centroids in the comparison between the PC group and the PC met group. Interestingly, at the species level, patients without metastases exhibited lower Z-scores at centroid one and higher Z-scores at centroid two (Figure 2d). Moreover, a scatter plot of the Z-scores computed within each cluster showed that the two species clusters clearly discriminated PC from PC met patients (Figure 2d). The heatmaps in Figure 3 show the relative abundance of the bacterial taxa composing each cluster at the phylum (A), family (B), genus (C) and species (D) levels for each study participant.

Gut microbiota composition (i.e. mean relative abundance %) at phylum (a), family (b), genus (c), and species (d) levels in patients with pancreas tumor without (PC) and with metastases (PC met) at the enrollment. The “Others” category includes all bacteria whose mean relative abundance is less than 1% (at phylum and family level), 0.5% (at genus level) and 0.1% (at species level), respectively. As expected, the percentage of bacteria included in the “Others” category tends to increase as more specific taxa levels are considered.

Box plots of the Z-scores computed within each cluster (i.e. centroid) detected by PEnalized LOgistic Regression Analysis at phylum (a), family (b), genus (c), and species (D1) taxonomic hierarchy. As two different clusters of bacteria population were detected at species level, a scatter plot of the Z-scores computed within the two centroids was also shown (D2). Points depicted into the scatterplot represent the Z-scores pair, computed at each individual patient, and were filled with blue and red colors to denote PC and PC met patients (groups), respectively. Moreover, a polygon connecting the outermost data points is shown for each group to aid the visualization.

Heatmaps of relative abundance (%) of bacterial populations identified by the PEnalized LOgistic Regression Analysis within each cluster at different taxonomic hierarchy. The order of the rows is determined by the main heatmap, which has been defined at the phylum level. All other heatmaps are automatically adjusted according to the settings in the main heatmap.

**Table 2.** Results from PEnalized LOgistic Regression Analysis (PELORA) in patients with PC without and with metastases.

Taxa level	Cluster number	Selected bacteria (within each cluster)	Quantity	Statistics	PC (*N* = 25)	PC met (*N* = 28)	*p*-value^#^
Phylum	1	Tenericutes	Relative abundance (%)	Mean ± SD	0.018 (0.064)	1.178 (3.784)	—
				Median [IQR]	0.003 [0.002, 0.009]	0.004 [0.003, 0.051]	
			Z-score°	Mean ± SD	−0.288 (0.543)	0.257 (1.232)	.047
		Bacteroidetes	Relative abundance (%)	Mean ± SD	43.980 (15.421)	46.013 (14.275)	—
				Median [IQR]	45.795 [38.235, 50.145]	48.918 [35.411, 54.319]	
			Z-score°	Mean ± SD	−0.104 (1.237)	0.093 (0.739)	.481
		Nitrospinae	Relative abundance (%)	Mean ± SD	0.000 (0.001)	0.006 (0.026)	—
				Median [IQR]	0.000 [0.000, 0.000]	0.000 [0.000, 0.000]	
			Z-score°	Mean ± SD	−0.171 (0.627)	0.152 (1.235)	.244
		Cluster centroid	Z-score (means)	Mean ± SD	−0.187 (0.484)	0.167 (0.623)	.026
Family	1	Anaeroplasmataceae	Relative abundance (%)	Mean ± SD	0.001 (0.001)	0.106 (0.361)	—
				Median [IQR]	0.000 [0.000, 0.001]	0.000 [0.000, 0.003]	
			Z-score°	Mean ± SD	−0.263 (0.409)	0.235 (1.287)	.070
		Sutterellaceae	Relative abundance (%)	Mean ± SD	2.481 (2.897)	2.938 (2.684)	—
				Median [IQR]	1.592 [1.214, 2.148]	1.957 [1.231, 3.984]	
			Z-score°	Mean ± SD	−0.207 (1.230)	0.185 (0.711)	.155
		Methanomassiliicoccaceae	Relative abundance (%)	Mean ± SD	Absent	0.016 (0.058)	—
				Median [IQR]		0.000 [0.000, 0.000]	
			Z-score°	Mean ± SD	Absent	0.193 (1.358)	.052^§^
		Pasteurellaceae	Relative abundance (%)	Mean ± SD	0.064 (0.085)	0.173 (0.242)	—
				Median [IQR]	0.020 [0.006, 0.113]	0.062 [0.007, 0.214]	
			Z-score°	Mean ± SD	−0.205 (0.905)	0.183 (1.060)	.161
		Porphyromonadaceae	Relative abundance (%)	Mean ± SD	3.405 (3.460)	4.438 (3.367)	—
				Median [IQR]	2.146 [1.520, 3.484]	3.255 [1.775, 7.025]	
			Z-score°	Mean ± SD	−0.171 (1.023)	0.153 (0.972)	.243
		Lactobacillaceae	Relative abundance (%)	Mean ± SD	1.417 (3.578)	2.628 (8.473)	—
				Median [IQR]	0.120 [0.049, 0.337]	0.147 [0.057, 0.898]	
			Z-score°	Mean ± SD	−0.064 (0.945)	0.058 (1.061)	.662
		Oscillospiraceae	Relative abundance (%)	Mean ± SD	1.207 (1.193)	1.576 (1.183)	—
				Median [IQR]	1.124 [0.472, 1.352]	1.347 [0.644, 2.232]	
			Z-score°	Mean ± SD	−0.206 (1.159)	0.184 (0.811)	.158
		Bacteroidaceae	Relative abundance (%)	Mean ± SD	25.743 (13.190)	26.615 (11.469)	—
				Median [IQR]	26.584 [16.683, 31.702]	28.312 [21.088, 30.824]	
			Z-score°	Mean ± SD	−0.097 (1.229)	0.087 (0.753)	.510
		Enterococcaceae	Relative abundance (%)	Mean ± SD	0.307 (0.663)	1.209 (5.605)	—
				Median [IQR]	0.057 [0.033, 0.192]	0.056 [0.029, 0.135]	
			Z-score°	Mean ± SD	0.013 (0.914)	−0.011 (1.087)	.932
		Fusobacteriaceae	Relative abundance (%)	Mean ± SD	0.174 (0.790)	0.223 (0.875)	—
				Median [IQR]	0.006 [0.001, 0.021]	0.007 [0.003, 0.041]	
			Z-score°	Mean ± SD	−0.173 (1.013)	0.155 (0.980)	.237
		Morganellaceae	Relative abundance (%)	Mean ± SD	0.039 (0.074)	0.097 (0.300)	—
				Median [IQR]	0.018 [0.009, 0.034]	0.014 [0.007, 0.041]	
			Z-score°	Mean ± SD	−0.057 (0.929)	0.051 (1.074)	.698
		Xanthomonadaceae	Relative abundance (%)	Mean ± SD	0.003 (0.002)	0.021 (0.098)	—
				Median [IQR]	0.002 [0.002, 0.003]	0.003 [0.002, 0.004]	
			Z-score°	Mean ± SD	−0.140 (0.622)	0.125 (1.244)	.339
		Cluster centroid	Z-score (means)	Mean ± SD	−0.149 (0.265)	0.133 (0.241)	<.001
Genus	1	Provencibacterium	Relative abundance (%)	Mean ± SD	0.003 (0.008)	0.000 (0.000)	—
				Median [IQR]	0.000 [0.000, 0.000]	0.000 [0.000, 0.000]	
			Z-score°	Mean ± SD	0.309 (1.356)	−0.275 (0.354)	.032
		Porphyromonas	Relative abundance (%)	Mean ± SD	0.032 (0.023)	0.022 (0.015)	—
				Median [IQR]	0.030 [0.015, 0.037]	0.018 [0.013, 0.029]	
			Z-score°	Mean ± SD	0.302 (0.875)	−0.270 (1.042)	.036
		Raoultella	Relative abundance (%)	Mean ± SD	0.016 (0.056)	0.002 (0.003)	—
				Median [IQR]	0.001 [0.000, 0.007]	0.001 [0.000, 0.002]	
			Z-score°	Mean ± SD	0.224 (1.267)	−0.200 (0.641)	.125
		Pseudoramibacter	Relative abundance (%)	Mean ± SD	0.005 (0.024)	0.000 (0.000)	—
				Median [IQR]	0.000 [0.000, 0.000]	0.000 [0.000, 0.000]	
			Z-score°	Mean ± SD	0.203 (1.398)	−0.181 (0.340)	.164
		Kluyvera	Relative abundance (%)	Mean ± SD	0.015 (0.071)	0.000 (0.000)	—
				Median [IQR]	0.000 [0.000, 0.000]	0.000 [0.000, 0.000]	
			Z-score°	Mean ± SD	0.203 (1.435)	−0.182 (0.151)	.164
		Slackia	Relative abundance (%)	Mean ± SD	0.016 (0.033)	0.004 (0.005)	—
				Median [IQR]	0.003 [0.001, 0.009]	0.001 [0.000, 0.005]	
			Z-score°	Mean ± SD	0.248 (1.152)	−0.221 (0.799)	.088
		Leptotrichia	Relative abundance (%)	Mean ± SD	0.012 (0.037)	0.002 (0.005)	—
				Median [IQR]	0.001 [0.000, 0.004]	0.000 [0.000, 0.002]	
			Z-score°	Mean ± SD	0.202 (1.183)	−0.180 (0.781)	.167
		Cluster centroid	Z-score (means)	Mean ± SD	0.241 (0.471)	−0.216 (0.288)	<.001
Species	1	Coprobacter secundus	Relative abundance (%)	Mean ± SD	0.009 (0.040)	0.056 (0.122)	—
				Median [IQR]	0.000 [0.000, 0.001]	0.004 [0.000, 0.035]	
			Z-score°	Mean ± SD	−0.418 (0.626)	0.373 (1.128)	.003
		Turicibacter sanguinis	Relative abundance (%)	Mean ± SD	0.000 (0.001)	0.011 (0.043)	—
				Median [IQR]	0.000 [0.000, 0.000]	0.000 [0.000, 0.002]	
			Z-score°	Mean ± SD	−0.319 (0.460)	0.284 (1.249)	.027
		Phascolarctobacterium succinatutens	Relative abundance (%)	Mean ± SD	0.003 (0.014)	0.226 (0.719)	—
				Median [IQR]	0.000 [0.000, 0.001]	0.000 [0.000, 0.002]	
			Z-score°	Mean ± SD	−0.281 (0.472)	0.251 (1.261)	.052
		Bacteroides thetaiotaomicron	Relative abundance (%)	Mean ± SD	0.251 (0.370)	0.661 (0.995)	—
				Median [IQR]	0.113 [0.023, 0.197]	0.318 [0.117, 0.873]	
			Z-score°	Mean ± SD	−0.346 (0.960)	0.309 (0.947)	.016
		Pseudomonas aeruginosa	Relative abundance (%)	Mean ± SD	0.001 (0.001)	0.015 (0.064)	—
				Median [IQR]	0.000 [0.000, 0.001]	0.000 [0.000, 0.002]	
			Z-score°	Mean ± SD	−0.284 (0.436)	0.254 (1.271)	.049
		Intestinimonas timonensis	Relative abundance (%)	Mean ± SD	0.001 (0.001)	0.009 (0.029)	—
				Median [IQR]	0.001 [0.000, 0.001]	0.001 [0.000, 0.003]	
			Z-score°	Mean ± SD	−0.290 (0.443)	0.259 (1.267)	.045
		Lactobacillus gasseri	Relative abundance (%)	Mean ± SD	0.038 (0.118)	0.073 (0.163)	—
				Median [IQR]	0.000 [0.000, 0.009]	0.001 [0.000, 0.062]	
			Z-score°	Mean ± SD	−0.142 (0.880)	0.127 (1.096)	.333
		Blautia massiliensis	Relative abundance (%)	Mean ± SD	Absent	0.009 (0.039)	—
				Median [IQR]		0.000 [0.000, 0.000]	
			Z-score°	Mean ± SD	Absent	0.179 (1.362)	.095^§^
		Parabacteroides sp. S448	Relative abundance (%)	Mean ± SD	0.022 (0.109)	0.033 (0.124)	—
				Median [IQR]	0.000 [0.000, 0.001]	0.000 [0.000, 0.001]	
			Z-score°	Mean ± SD	−0.103 (0.895)	0.092 (1.093)	.483
		Haemophilus parainfluenzae	Relative abundance (%)	Mean ± SD	0.001 (0.004)	0.016 (0.073)	—
				Median [IQR]	0.000 [0.000, 0.000]	0.000 [0.000, 0.000]	
			Z-score°	Mean ± SD	−0.168 (0.562)	0.150 (1.263)	.252
		Fournierella massiliensis	Relative abundance (%)	Mean ± SD	0.001 (0.001)	0.019 (0.084)	—
				Median [IQR]	0.000 [0.000, 0.002]	0.001 [0.000, 0.002]	
			Z-score°	Mean ± SD	−0.186 (0.492)	0.166 (1.285)	.205
		Parabacteroides sp. SN4	Relative abundance (%)	Mean ± SD	0.003 (0.007)	0.055 (0.249)	—
				Median [IQR]	0.000 [0.000, 0.001]	0.001 [0.000, 0.004]	
			Z-score°	Mean ± SD	−0.251 (0.737)	0.224 (1.154)	.084
		Butyricimonas paravirosa	Relative abundance (%)	Mean ± SD	0.008 (0.020)	0.019 (0.045)	—
				Median [IQR]	0.000 [0.000, 0.002]	0.000 [0.000, 0.003]	
			Z-score°	Mean ± SD	−0.052 (0.819)	0.047 (1.151)	.723
		Shimwellia blattae	Relative abundance (%)	Mean ± SD	0.008 (0.038)	0.000 (0.000)	—
				Median [IQR]	0.000 [0.000, 0.000]	0.000 [0.000, 0.000]	
			Z-score°	Mean ± SD	0.018 (1.469)	−0.016 (0.077)	.901
		Clostridium lactatifermentans	Relative abundance (%)	Mean ± SD	0.008 (0.029)	0.011 (0.044)	—
				Median [IQR]	0.002 [0.001, 0.003]	0.001 [0.000, 0.004]	
			Z-score°	Mean ± SD	0.021 (0.963)	−0.019 (1.049)	.886
		Cluster centroid	Z-score (means)	Mean ± SD	−0.181 (0.099)	0.162 (0.152)	<.001
	2	Eubacterium ventriosum	Relative abundance (%)	Mean ± SD	0.073 (0.203)	0.018 (0.032)	—
				Median [IQR]	0.011 [0.000, 0.074]	0.001 [0.000, 0.021]	
			Z-score°	Mean ± SD	0.213 (1.103)	−0.190 (0.875)	.145
		Raoultella ornithinolytica	Relative abundance (%)	Mean ± SD	0.008 (0.023)	0.001 (0.002)	—
				Median [IQR]	0.000 [0.000, 0.003]	0.000 [0.000, 0.001]	
			Z-score°	Mean ± SD	0.305 (1.290)	−0.272 (0.534)	.035
		Bacteroides sp. Marseille-P3108	Relative abundance (%)	Mean ± SD	0.190 (0.928)	0.151 (0.778)	—
				Median [IQR]	0.004 [0.002, 0.007]	0.004 [0.002, 0.005]	
			Z-score°	Mean ± SD	0.065 (0.997)	−0.058 (1.018)	.657
		Clostridium disporicum	Relative abundance (%)	Mean ± SD	Absent	0.013 (0.048)	—
				Median [IQR]		0.000 [0.000, 0.000]	
			Z-score°	Mean ± SD	Absent	0.168 (1.365)	.095^§^
		Veillonella sp. 2011 Oral VSA C3	Relative abundance (%)	Mean ± SD	0.013 (0.063)	0.000 (0.000)	—
				Median [IQR]	0.000 [0.000, 0.000]	0.000 [0.000, 0.000]	
			Z-score°	Mean ± SD	0.154 (1.456)	−0.137 (0.000)	.294
		Bacteroides eggerthii	Relative abundance (%)	Mean ± SD	0.068 (0.194)	0.003 (0.004)	—
				Median [IQR]	0.002 [0.000, 0.009]	0.001 [0.000, 0.004]	
			Z-score°	Mean ± SD	0.254 (1.254)	−0.227 (0.644)	.080
		Prevotella oris	Relative abundance (%)	Mean ± SD	0.008 (0.027)	0.035 (0.177)	—
				Median [IQR]	0.001 [0.000, 0.004]	0.000 [0.000, 0.002]	
			Z-score°	Mean ± SD	0.117 (0.956)	−0.104 (1.044)	.428
		Prevotella sp. 109	Relative abundance (%)	Mean ± SD	0.784 (1.625)	0.495 (1.621)	—
				Median [IQR]	0.008 [0.003, 0.421]	0.005 [0.002, 0.025]	
			Z-score°	Mean ± SD	0.169 (1.073)	−0.151 (0.923)	.248
		Bifidobacterium boum	Relative abundance (%)	Mean ± SD	0.000 (0.000)	0.000 (0.000)	—
				Median [IQR]	0.000 [0.000, 0.000]	0.000 [0.000, 0.000]	
			Z-score°	Mean ± SD	−0.154 (1.456)	0.137 (0.000)	.294
		Romboutsia sedimentorum	Relative abundance (%)	Mean ± SD	0.000 (0.000)	0.016 (0.082)	—
				Median [IQR]	0.000 [0.000, 0.000]	0.000 [0.000, 0.000]	
			Z-score°	Mean ± SD	−0.167 (0.000)	0.149 (1.370)	.254
		Parabacteroides distasonis	Relative abundance (%)	Mean ± SD	0.857 (1.054)	0.822 (1.124)	—
				Median [IQR]	0.560 [0.242, 0.873]	0.294 [0.111, 1.017]	
			Z-score°	Mean ± SD	0.154 (0.860)	−0.137 (1.108)	.295
		Prevotella multisaccharivorax	Relative abundance (%)	Mean ± SD	0.202 (1.007)	0.001 (0.002)	—
				Median [IQR]	0.000 [0.000, 0.001]	0.000 [0.000, 0.000]	
			Z-score°	Mean ± SD	0.100 (1.394)	−0.090 (0.426)	.496
		Desulfovibrio legallii	Relative abundance (%)	Mean ± SD	0.010 (0.050)	0.000 (0.002)	—
				Median [IQR]	0.000 [0.000, 0.000]	0.000 [0.000, 0.000]	
			Z-score°	Mean ± SD	0.085 (1.338)	−0.076 (0.568)	.565
		Eikenella corrodens	Relative abundance (%)	Mean ± SD	0.000 (0.002)	0.004 (0.021)	—
				Median [IQR]	0.000 [0.000, 0.000]	0.000 [0.000, 0.000]	
			Z-score°	Mean ± SD	−0.023 (0.636)	0.020 (1.251)	.877
		Clostridium sp. 14505	Relative abundance (%)	Mean ± SD	0.052 (0.111)	0.007 (0.020)	—
				Median [IQR]	0.000 [0.000, 0.024]	0.000 [0.000, 0.001]	
			Z-score°	Mean ± SD	0.301 (1.184)	−0.269 (0.722)	.037
		Unknown	Relative abundance (%)	Mean ± SD	65.723 (9.592)	64.408 (7.335)	—
				Median [IQR]	62.599 [60.553, 71.801]	64.064 [57.419, 69.453]	
			Z-score°	Mean ± SD	0.110 (1.236)	−0.098 (0.740)	.454
		Cluster centroid	Z-score (means)	Mean ± SD	0.093 (0.168)	−0.083 (0.239)	.003

Abbreviations: PC: patients with pancreatic tumor without metastasis at enrollment; PC met: patients with pancreatic tumor with metastasis at enrollment; IQR: Interquartile range (i.e. first-third quartiles); SD: Standard Deviation; Absent: all values are 0%. Standardized Z-score: As a first step, the relative abundance (%) of each bacterium was logit transformed (so that values can theoretically range from negative to positive infinity) and, as a second step, the Z-score was computed by standardizing the transformed variable (i.e., taking the variable values, subtracting its mean and dividing by its SD). The centroid is calculated as the average Z-score of all the bacteria within each cluster. ^#^All p-values were derived from the parametric two-sample t-test on Z-scores, with the exception of those marked as “^§^”, which were derived from the Mann-Whitney U test. The latter was performed in presence of no variance in one of the two groups (i.e., when the group has all values equals to 0% - denoted as “Absent”.

### Machine learning approach to identify microbial patterns predictive of metastases

**Figure 4.**
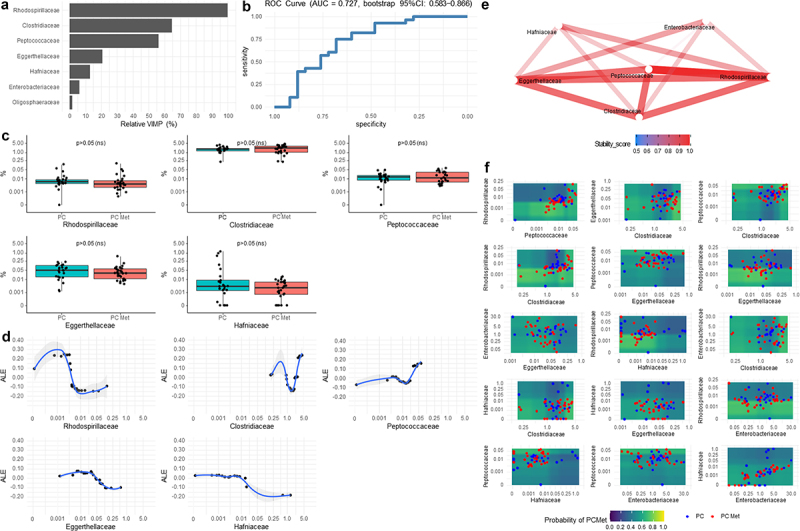
Results from iterative Random Forest of the most important bacteria detected at family level.

**Figure 5.**
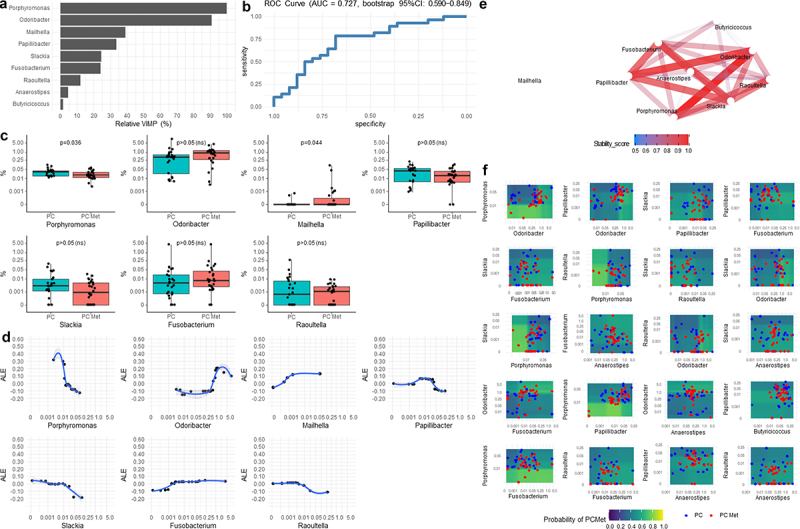
Results from iterative Random Forest of the most important bacteria detected at genus level.

**Figure 6.**
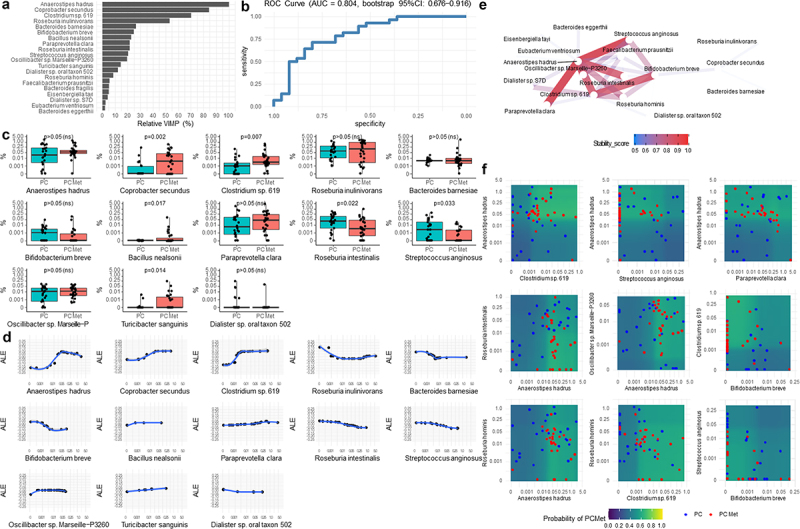
Results from iterative random forest of the most important bacteria detected at species level.

Results from iRFs at the family, genus, and species level are shown in Figures 4–6, respectively. The optimal number of iterations as well as the regularization factor for each iRF were detected in the “tuning phase” as shown in Supplemental statistical methods.

(a) Variable importance plot is rescaled from 0% to 100% (relative VIMP) with respect to the maximum achieved value. Only variables with VIMP > 0 are shown and ranked from the most to the less important. (b) ROC curve showing the sensitivity and specificity achieved at each possible cut-off of the predicted probabilities computed by the iRF from the out-of-bag data, and hence is not prone to overfitting. The area under the ROC curve (AUC) was corroborated by its 95% confidence interval, computed after 1000 stratified bootstrap replicates. The AUC quantifies the discriminatory power achieved by the iRF, which reflect the one achieved by the all variables reported in the VIMP plot. (c) Boxplots of the relative abundance (%) of all variables detected by the iRF with VIMP > 10%. (d) Accumulated Local Effect (ALE) of all variables detected by the iRF with VIMP > 10%. ALE describes how the variables influence the predicted probability on average. The gray band is a confidence band for the regression line fitted in the estimated ALE points. (e) Network plot of the most stable two-order interactions (i.e. with a stability score ≥0.5). The stability of a recovered interaction is defined as the proportion of times that interaction appears as an output of the generalized Random Intersection Trees, after a bootstrap perturbation of the data (i.e. 0 = totally instable interaction,1=totally stable interaction). The higher the stability score, the better is the quality of the recovered interaction. (f) Partial Dependence Plot (PDP) produced for variables with top stable interactions (i.e. stability score >0.70). PDPs show the marginal (total) effect that two variables have on the predicted outcome. Colored zones locate those regions at which the metastatic event more likely occurs (green/yellow) and not occurs (blue/violet). Individual observations are plotted with respect to each variable combination. Relative abundances (%) values reported in c, d and f panels are in logit scale.

(a) Variable importance plot is rescaled from 0% to 100% (relative VIMP) with respect to the maximum achieved value. Only variables with VIMP > 0 are shown and ranked from the most to the less important. (b) ROC curve showing the sensitivity and specificity achieved at each possible cut-off of the predicted probabilities computed by the iRF from the out-of-bag data, and hence is not prone to overfitting. The Area under the ROC curve (AUC) was corroborated by its 95% confidence interval, computed after 1000 stratified bootstrap replicates. The AUC quantifies the discriminatory power achieved by the iRF, which reflect the one achieved by the all variables reported in the VIMP plot. (c) Boxplots of the relative abundance (%) of all variables detected by the iRF with VIMP > 10%. (d) Accumulated Local Effect (ALE) of all variables detected by the iRF with VIMP > 10%. ALE describes how the variables influence the predicted probability on average. The gray band is a confidence band for the regression line fitted in the estimated ALE points. (e) Network plot of the most stable two-order interactions (i.e. with a stability score ≥0.5). The stability of a recovered interaction is defined as the proportion of times that interaction appears as an output of the generalized Random Intersection Trees, after a bootstrap perturbation of the data (i.e. 0 = totally instable interaction,1=totally stable interaction). The higher the stability score, the better is the quality of the recovered interaction. (f) Partial Dependence Plot (PDP) produced for variables with top stable interactions (i.e. stability score >0.70). PDPs show the marginal (total) effect that two variables have on the predicted outcome. Colored zones locate those regions at which the metastatic event more likely occurs (green/yellow) and not occurs (blue/violet). Individual observations are plotted with respect to each variable combination. Relative abundances (%) values reported in c, d and f panels are in logit scale.

(a) Variable importance plot is rescaled from 0% to 100% (relative VIMP) with respect to the maximum achieved value. Only variables with VIMP > 0 are shown and ranked from the most to the less important. (b) ROC curve showing the sensitivity and specificity achieved at each possible cut-off of the predicted probabilities computed by the iRF from the out-of-bag data, and hence is not prone to overfitting. The Area under the ROC curve (AUC) was corroborated by its 95% confidence interval, computed after 1000 stratified bootstrap replicates. The AUC quantifies the discriminatory power achieved by the iRF, which reflect the one achieved by the all variables reported in the VIMP plot. (c) Boxplots of the relative abundance (%) of all variables detected by the iRF with VIMP > 10%. (d) Accumulated Local Effect (ALE) of all variables detected by the iRF with VIMP > 10%. ALE describes how the variables influence the predicted probability on average. The gray band is a confidence band for the regression line fitted in the estimated ALE points. (e) Network plot of the most stable two-order interactions (i.e. with a stability score ≥0.5). The stability of a recovered interaction is defined as the proportion of times that interaction appears as an output of the generalized Random Intersection Trees, after a bootstrap perturbation of the data (i.e. 0 = totally instable interaction,1=totally stable interaction). The higher the stability score, the better is the quality of the recovered interaction. (f) Partial Dependence Plot (PDP) produced for variables with top stable interactions (i.e. stability score >0.70). PDPs show the marginal (total) effect that two variables have on the predicted outcome. Colored zones locate those regions at which the metastatic event more likely occurs (green/yellow) and not occurs (blue/violet). Individual observations are plotted with respect to each variable combination. Relative abundances (%) values reported in c, d and f panels are in logit scale.

In the Variable Importance (VIMP) plot (Figure 4a), the families *Rhodospirillaceae*, *Clostridiaceae*, *Peptococcaceae*, *Eggerthellaceae*, *Hafniaceae* were found to provide the highest contribution (i.e., Relative VIMP > 10%) in detecting the presence of metastases, while *Enterobacteriaceae* and *Oligosphaeraceae* provided the least contribution to this discrimination. Sensitivity and specificity achieved at each possible cutoff of the predicted probabilities (of having metastases) computed by the iRF from the out-of-bag data were shown in the ROC curve (Figure 4b), in which AUC value of 0.727 (bootstrap 95% CI: 0.583–0.866) means that taking a metastatic PC patient and a non-metastatic PC patient at random, the probability that the algorithm assigns a higher probability of metastases in the metastatic patient (with respect to the non-metastatic patient) is 72.7%, although in terms of relative abundance none of the families showed statistically significant variations in the two groups (Figure 4c). ALE was estimated from iRF on the most important variables with the aim of showing the relationship between the relative abundance levels of specific bacteria and the mean predicted probability of having metastases with respect to the overall mean predicted probability defined at ALE = 0 (i.e., average risk). Specifically, the *Rhodospirillaceae* family showed a sigmoid curve in which lower levels (0–0.001%) were associated with a higher probability of having metastases (ALE = 0.30, i.e. 30% higher than the average risk), whereas higher levels (0.01–0.25%) were associated with a lower probability of metastases (ALE = −0.10, i.e. 10% lower than the average risk). A non-linear and non-monotonic curve was assumed for the *Clostridiaceae* family, in which the probability of metastases decreased within the 1.0–2.5% range, whereas it was higher above and below this interval. A near linear curve was also observed for *Peptococcaceae*, for which higher abundance correlated with a higher probability of metastases, and for *Eggerthellaceae* and *Hafniaceae*, for which increased levels predicted a lower probability of metastases, as compared to the average risk (Figure 4d). The network plot of interactions with the highest stability among the families is graphically represented in Figure 4e (stability score >0.50), with the main interactions involving *Rhodospirillaceae*, *Peptococcaceae*, *Eggerthellaceae* and *Clostridiaceae*. The aforementioned dual interactions were integrated into the PDPs (Figure 4f), highlighting the regions in which patients with metastases were more likely to be found. Remarkably, patients with *Rhodospirillaceae* abundance within the range of 0.00031–0.003% together with *Peptococcaceae* within 0.002–0.008%, *Clostridiaceae* within 0.19–2.60%, *Eggerthellaceae* within 0.001–0.042%, *Hafniaceaea* <0.01%, *Enterobacteriaceae* <30.4% showed a 70–80% probability of having metastases at the time of enrollment. At the genus level, *Porphyromonas* and *Odoribacter* provided the highest contribution to detecting metastases, followed by *Mailhella*, *Papillibacter*, *Slackia*, *Fusobacterium* and *Raoultella* whereas *Anaerostipes* and *Butyricicoccus* contributed the least (Figure 5a). In Figure 5b, the ROC curve is reported, which denoted a fair quality level of the iRF discrimination ability: AUC = 0.727 (bootstrap 95% CI: 0.590–0.849). The relative abundance of *Porphyromonas* and *Mailhella* genera was significantly decreased for the former and enriched for the latter in the PC met group compared the PC group (*p* = .036 and *p* = .044, respectively) (Figure 5c). Regarding ALE plots, one of the lowest *Porphyromonas* levels (close to 0.005%) was associated with a higher probability of metastases (ALE = 0.30, i.e. 30% higher than the average risk probability). Non-monotonic curves were found for the *Odoribacter* and *Papillibacter*. For *Mailhella* and *Fusobacterium*, an increase in the probability of metastasis was observed as the relative abundance plateaued. In contrast, for *Slackia* and *Raoultella* genera, after the initial plateau phase, the probability of metastases started to decrease slightly (Figure 5d). The most stable interaction pathways among the genera are shown in Figure 5e. At this taxonomic level, PDPs (Figure 5f) suggested that patients with *Porphyromonas* abundance lower than 0.01% together with *Odoribacter* <1.2%, *Raoultella* <0.01%, *Slackia* <0.02%, *Papillibacter* <0.025%, or *Fusobacterium* >0.001% showed approximately 70–80% probability of having metastases at the time of enrollment. Worth of note was also the case of patients with *Papillibacter* levels >0.001% and *Fusobacterium* levels between 0.005% and 0.03%, which have more than 60% probability of having metastases. A further interaction involving *Fusobacterium* was the one with *Anaerostipes*: *Fusobacterium* abundance higher than 0.001% together with *Anaerostipes* levels greater than 0.04% showed an approximately 55–60% probability of having metastases. At the species level, *Anaerostipes hadrus*, *Coprobacter secundus*, *Clostridium* sp. 619, and *Roseburia inulinivorans* were the most important, followed by *Bacteroides barnesiae*, *Bifidobacterium breve*, *Bacillus nealsonii*, *Paraprevotella clara*, *Roseburia intestinalis*, *Streptococcus anginosus, Oscillibacter* sp. Marseille-P3260, *Turicibacter sanguinis*, *Dialister* sp. Oral taxon 502 and seven other species with VIMP < 10% (Figure 6a). The ROC curve shown in Figure 6b shows a good quality level of iRF discriminatory ability: AUC = 0.804 (bootstrap 95% CI: 0.676–0.916). The relative abundance of *Coprobacter secundus* (*p* = .002), *Clostridium* sp. 619 (*p* = .007), *Bacillus nealsonii* (*p* = .017), and *Turicibacter sanguinis* (*p* = .014) showed a significant increase in the PC met group compared to the PC group, whereas *Roseburia intestinalis* (*p* = .022) and *Streptococcus anginosus* (*p* = .033) were increased in PC patients compared to PC met patients (Figure 6c). In the ALE plots, *Anaerostipes hadrus*, *Coprobacter secundus*, *Clostridium* sp. 619, and *Bifidobacterium breve* showed a sigmoid curve (Figure 6d). As for *Anaerostipes hadrus*, *Coprobacter secundus* and *Clostridium* sp. 619 metastases were less probable at lower levels and then became more probable as the relative abundance increased. In contrast, for *Bifidobacterium breve*, the probability of metastases was higher at lower abundance levels and then decreased when abundance increased. At this taxonomic level, it was interesting to note that the course of *Roseburia inulinivorans* represented by a U-shaped curve (Figure 6d). The most stable interaction pathways among the detected species are shown in Figure 6e. At this taxonomic level, PDPs (Figure 6f) suggested that patients with *Anaerostipes hadrus* abundance greater than 0.032% together with *Clostridium* sp. 619 > 0.002% or *Roseburia intestinalis <*0.008%, and patients with Clostridium sp. 619 > 0.002% together with *Bifidobacterium breve* <0.001% showed approximately 60–65% probability of having metastases at the time of enrollment.

At all taxonomic levels, it should be noted that the ALE plots were performed on all variables detected by the iRF with VIMP > 10%, whereas the PDPs were produced only for those variables with stable interactions (i.e., with stability score >0.70).

### Functional prediction of gut microbial profiles

**Figure 7.**
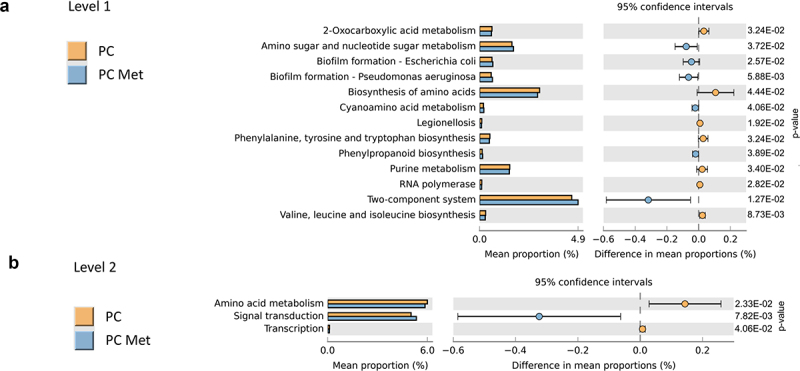
Functional prediction for gut microbial populations in PC and PC met patients.

In order to get a more comprehensive view of the role of gut microbiota in PC metastatization, we performed a prediction of the functional/metabolic capabilities of the microbial communities identified by 16S rRNA sequencing in the two experimental groups, by using the software package Tax4Fun2. As shown in Figure 7, a prediction of bacterial functions at level 1 (A) and 2 (B) of KEGG pathways was performed. Among the main differences, the biosynthesis and metabolism of amino acids (above all aromatic and branched chain amino acids) and the transcriptions function were increased in PC patients, whereas signal transduction was increased in PC met.

Significant KEGG pathways for gut microbiota between PC and PC met patients at level 1 (a) and 2 (b), respectively. Pathways were considered significant when the *p*-value from Mann–Whitney test was <0.05.

### Fecal metabolomics analysis

**Figure 8.**
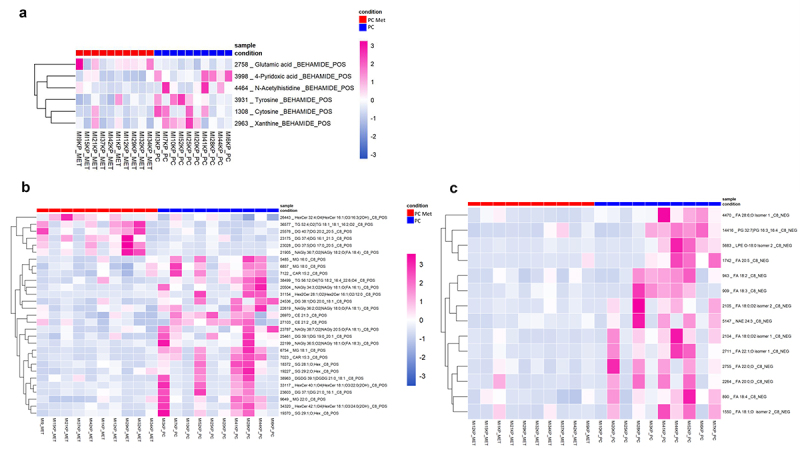
Fecal metabolic profile in PC and PC met patients.

In addition to the functional profile, a metabolomics analysis on fecal samples of a subset of patients from each of the two groups was carried out. Figure 8 shows the heatmaps representing the 6 out of 106 polar metabolites (A), the 29 out 545 lipids identified in positive ionization mode (B) and the 14 out of 317 lipids identified in negative ionization mode (C) that were significantly different among the two groups, respectively. As for polar compounds (Figure 8a), glutamic acid was found to be significantly enriched in PC met patients, whereas 4-pyridoxic acid, N-acetylhistidine, tyrosine, cytosine and xanthine were found increased in non-metastatic PC patients. A greater impact was observed on lipid metabolism, in which the most relevant classes found in positive polarity were diacylglycerols (DG) and N-acyl glycines (NAGly). Some DG were found enriched in metastatic patients (DG 40:7|DG 20:2_20:5, DG 37:4|DG 16:1_21:3 and DG 37:5|DG 17:0_20:5) while others were increased in non-metastatic patients (DG 39:1|DG 19:0_20:1, DG 38:1|DG 20:0_18:1 and DG 37:1|DG 21:0_16:1). Instead, N-acyl glycines were statistically more enriched in non-metastatic patients (e.g., NAGly 38:7;O2|NAGly 20:5;O(FA 18:1), NAGly 36:5;O2|NAGly 18 :1;O(F 18:3), NAGly 34:3;O2|NAGly 18:1;O(F 16:1), NAGly 36:7;O2|NAGly 18:2;O(F 18:4), NAGly 36:2;O2|NAGly 18:0;O(FA 18:1)). However, the most significant impact on intestinal lipid composition was observed in negative polarity, where a class of oxidized fatty acids delineated a clear separation between the metastatic and non-metastatic groups, being enriched in the latter. This class includes FA 18:2, FA 18:3 and FA 20:5, known, respectively, as linolic acid, linolenic acid and 5,8,11,14,17-eicosapentaenoic acid (EPA) (Figure 8b).

Heatmaps showing the significantly different (t-test, *p* < .05) metabolites between PC (blue squares) and PC met (red squares) patients. Polar metabolites (a) and lipidic metabolites obtained in positive polarity (b) and negative polarity (c) are shown, respectively. Shades of colors from blue to pink represent the abundance of each compound.

### Correlations between gut microbiota and the fecal metabolome

**Figure 9.**
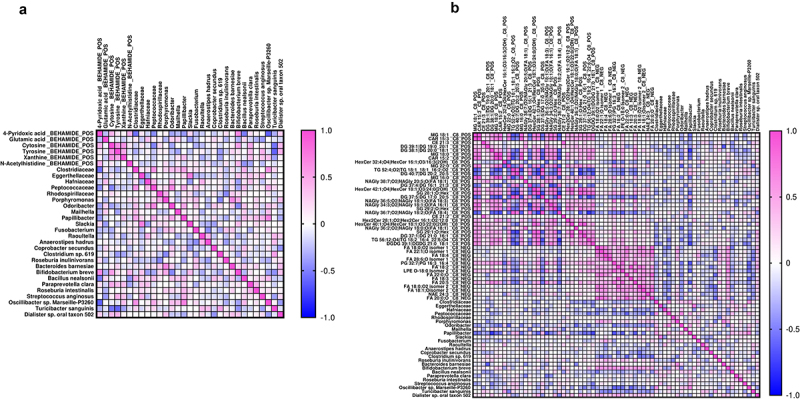
Associations between gut microbiota and the fecal metabolome.

Both the polar and the lipidic metabolites which were differently represented in PC and PC met were then correlated with the bacteria which best discriminate the two microbial profiles (Figure 9a, b, respectively). Regarding the polar metabolites, 4-pyridoxic acid showed a significant negative correlation with *Clostridiaceae* (*r*= − 0.56), *Peptococcaceae* (*r*= − 0.46), *Bacillus nealsonii* (*r*= − 0.52), *Oscillibacter* sp. Marseille-P3260 (*r*= − 0.62) and a significant correlation with *Bifidobacterium breve* (r = 0.67); cytosine was negatively associated with *Peptococcaceae* (*r*= − 0.49) and *Turicibacter sanguinis* (*r* = − 0.55) and positively associated with *Bifidobacterium breve* (r = 0.55); tyrosine was negatively correlated with and positively correlated with *Turicibacter sanguinis* (*r*= − 0.61) and positively associated with *Streptococcus anginosus* (*r* = 0.45). Concerning lipids, a considerable number of both positive and negative correlations were observed. Remarkably, at the family level, *Peptococcaceae* exhibited a significant negative correlation with FA 22:0;O (r = −0.61). At the genus level, *Papillibacter* was negatively correlated with DG 38:1|DG 20:0_18:1 (r = −0.77), NAGly 36:5;O2|NAGly 18:1;O(FA 18:3) (r = −0.68), DGDG 39:1|DGDG 21:0_18:1 (r = −0.72), PG 32:7|PG 16:3_16:4 (r = −0.67), LPE O-18:0 Isomer 2 (r = −0.62), and FA 20:5 (r = −0.63). At the species level, *Bacteroides barnesiae* and *Oscillibacter* sp. Marseille-P3260 showed significant negative correlations with DG 39:1|DG 19:0_20:1 (r = −0.65) and PG 32:7|PG 16:3_16:4 (r = −0.61), respectively. Finally, *Bifidobacterium breve* alone exhibited positive correlations with FA 18:0;O2 isomer 1 (r = 0.61), FA 22:1;O isomer 1 (r = 0.61), PG 32:7|PG 16:3_16:4 (r = 0.65), LPE O-18:0 Isomer 2 (r = 0.73), and FA 20:5 (r = 0.61).

Spearman’s rank correlations of the quantities of the significantly different polar metabolites (a) and lipids (b) from fecal samples with the intestinal bacteria discriminating the two groups of patients. Shades of colors from blue (negative correlation) to pink (positive correlation) indicate the value of the R correlation coefficient.

## Discussion

In the current study, a cohort of 53 patients with PC was recruited; 28 of these patients were already metastatic at the time of diagnosis. As expected, the PC met group showed a more advanced disease (the vast majority of patients had stage IV cancer) and a higher mortality rate than non-metastatic PC patients. Interestingly, the two groups differed significantly in terms of jaundice and endoprosthesis use, which were both more frequent in non-metastatic patients. Liver injury caused by endoprosthesis positioning could explain why hepatic biomarkers such as bilirubin, AST, GGT and ALP were remarkably higher than those in metastatic patients.

Metastases in PC represent a huge burden because they prevent patients from accessing potentially curative surgery; therefore, deeply delving into the mechanisms behind this process remains an urgent need. Recent studies suggest that the microbiota may be involved in both cancer progression and metastasis, through its interaction with the host immune system and the production of bacterial metabolites and molecules.^14,18^ In particular, LPS constituting the cell wall of Gram-negative bacteria has been described to induce epithelial-mesenchymal transition^28,29^ and promote angiogenesis through its interaction with TLR-4 on the host cell membranes and VEGF expression.^19,30^ To our knowledge, this is the first report investigating gut microbiota composition in patients with metastatic and non-metastatic PC. First, the PELORA approach was used to identify patterns of bacteria at different taxonomic levels, identified by 16S sequencing, that best discriminate the two groups. At first glance, an overall increase in Gram-negative bacteria was observed in metastatic PC patients compared to that in non-metastatic ones. Indeed, the taxa whose abundance increased the most in metastatic subjects, such as *Tenericutes*, *Anaeroplasmataceae*, *Phascolarctobacterium succinatutens*, *Pseudomonas aeruginosa*, *Haemophilus parainfluenzae*, *Fournierella massiliensis*, *Parabacteroides* sp. SN4, were all Gram-negative bacteria, leading us to speculate that an increase in the LPS-TLR4-VEGF pathway could have occurred, promoting angiogenesis and subsequent metastases. To further dissect the potential use of gut microbes and their abundance and to improve their performance in the identification of PC patients at risk of metastases, a machine learning algorithm was also implemented. This approach identified bacterial signatures and reciprocal interactions with good discriminatory power between patients with metastatic and non-metastatic PC. Among the bacterial taxa identified by the machine learning, the genera *Porphyromonas* and *Fusobacterium* deserve particular attention given their well-established association with PC, as well as with colorectal cancer.^31–34^ Surprisingly, according to our analysis, the risk of metastasis decreased as abundance of *Porphyromonas* increased. On the contrary, lower abundances of *Fusobacterium* slightly decreased the probability of metastatic events, in good agreement with the literature, according to which *F. nucleatum* promotes metastases in colorectal^35^ and esophageal^36^ cancers and elicits migration in pancreatic cancer cell lines.^34^ At the species level, the machine learning algorithm detected *Anaerostipes hadrus*, *Roseburia inulinivorans* and *Roseburia intestinalis*, well-known producers of butyrate,^37,38^ which has been reported to inhibit pancreatic cancer cell invasion *in vitro*.^39^ Consistently, in our analysis, the abundance of *R. inulinivorans* and *R. intestinalis* was inversely correlated with the risk of metastases, whereas *A. hadrus* was not. However, previous studies, have shown that *A. hadrus* is overabundant in children with type 1 diabetes mellitus^40^ and worsens colitis in mice,^37^ suggesting that, although potentially beneficial in healthy conditions, this butyrate producer may be harmful in diseased subjects.^37^ Interestingly, *Bifidobacterium breve* higher abundance showed a protective effect against metastases. Although, to the best of our knowledge, no anti-metastatic action has been documented yet for this bacterium, *B. breve* is generally regarded as a beneficial probiotic. It showed anti-tumor properties in mice with oral carcinoma,^41^ and it was associated with increased progression-free survival in lung cancer patients.^42^ Finally, among the main species with discriminatory power between the two groups we also found *Streptococcus anginosus*, whose abundance was reduced in metastatic compared to non-metastatic patients. This result is in line with a previous *in vitro* study in which *S. anginosus* supernatant inhibited the proliferation, migration and invasion ability of oral squamous cell carcinoma.^43^ AI, in which machine learning represents a field, is already widely applied in different areas of pancreatic cancer, from diagnosis to prognosis, including the prediction of survival, recurrence, metastases, and response to treatment.^44^ In our cohort of patients, with this approach, it was possible to understand that microbial variations within the intestinal microbiota may contribute to development of metastases in PC patients. Further research could expand the potential applications of this intervention strategy and guide clinicians in discriminating between patients with metastatic and non-metastatic PC. Specifically, by considering not only a single microorganism but also bacterial patterns with already known interactions, important information could be obtained for the development of new therapeutic and/or integrative strategies in PC treatment, as well as indications for improving the diagnostic process. In this regard, it will also be appropriate to pay attention to the relative abundance of microorganisms, since even minimal variations could have completely different effects from those expected. This can pave the way for new diagnostic devices based on new algorithms to support clinicians in their diagnosis.

When metabolomics analysis was performed on fecal samples from a subset of PC and PC met patients, a number of statistically significant changes emerged. Glutamic acid was found significantly enriched in the fecal samples from metastatic patients. Interestingly, glutamate has been implicated in increasing pancreatic cancer cells migration and invasion through binding to specific receptors and activating Kras-MAPK signaling.^45^ Tyrosine increase in the feces from PC patients without metastasis was consistent with the functional prediction performed on gut microbiota, in which phenylalanine, tyrosine and tryptophan biosynthesis resulted increased as well. Moreover, higher fecal levels of xanthine in the same group were again in agreement with the functional profile, which predicted an increase in purine metabolism in PC patients as compared to PC met. Lipidomics showed a number of statistically significant lipids, capable of highlighting changes between the PC group and the PC met group. Among lipids obtained in positive polarity, a class of DGs was found enriched in patients with metastases. Indeed, DG appears to be involved in several cellular mechanisms such as motility, survival and cell proliferation and an imbalance in its homeostasis or in the functioning of its effectors, especially protein kinase C (PKC), seems to be involved in the progression and development of metastasis.^46^ In a study on triple-negative breast cancer, high expression of a diacylglycerol kinase ζ (DGKZ) was observed to promote metastasis *in vitro* and *in vivo*.^47^ On the contrary, NAGly were found statistically enriched in patients without metastases. Interestingly, reduced expression of the glycine N-acyltransferase (GLYAT) gene was correlated with increased cell proliferation and increased migratory properties of tumor cells, perhaps due to the activation of PI3K/AKT/Snail signaling, which induces epithelial-mesenchymal transition (EMT).^48^ The lipidomics analysis conducted in negative polarity highlights a notable increase in the fatty acid class in patients without metastases in agreement with the study by Luo et al.^49^ In detail, the level of 5,8,11,14,17-eicosapentaenoic acid (EPA), increased in PC group, was found statistically reduced in the tumor tissue of metastatic patients with colorectal cancer.^50^ Although the fecal metabolome is the result of both host and gut microbiota metabolism, a remarkable number of significant correlations between metabolites and gut bacteria were recorded. Strikingly, *B. breve* was the species most associated with the metabolomic differences observed. For instance, it positively correlated with 4-pyridoxic acid, a vitamin B6 metabolite, whose imbalance has been involved in tumor progression.^51^ Moreover *B. breve* also showed positive correlations with lipids mostly belonging to the fatty acids class. Some research has highlighted the antineoplastic role of fatty acids due to apoptotic induction in tumor cells or to the reduction of resistance to chemotherapy treatments.^52,53^ Furthermore, in a study conducted on patients with non-small-cell lung cancer and healthy individuals, *B. breve* was included among the intestinal bacteria capable of promoting progression-free survival in patients undergoing immunocombined chemotherapy.^42^ To date, further studies are required to investigate any interactions between intestinal bacteria and the metabolomic and lipidomic features that could characterize a pathophysiological condition. Therefore, since the gut microbiota is gaining an emerging role in the pathogenesis and development of cancer, we believe that investigating the microbiome to its full potential may open the way to new supportive approaches for the management of this devastating disease.

## List of abbreviations

AI: artificial intelligence
ALE: accumulated local effects
ALP: alkaline phosphatase
CA19–9: carbohydrate antigen 19–9
CEA: carcino-embryonic antigen
CK: creatine kinase
CRP: C-reactive protein
DG: diacylglycerol
DGKZ: diacylglycerol kinase ζ
EMT: epithelial-mesenchymal transition
EPA: 5,8,11,14,17-eicosapentaenoic acid
ESR: erythrocyte sedimentation rate
GLYAT: glycine N-acyltransferase
GGT: gamma-glutamyl transferase
IC: informed consent
iRF: iterative random forest
LPS: lipopolysaccharide
NAGly: N-acyl glycine
PC: pancreatic cancer
PDPs: partial dependence plot
PELORA: PEnalized LOgistic Regression Analysis
PKC: protein kinase C
PT: prothrombin time
PTT: partial thromboplastin time
TLR-4: toll-like receptor 4
TME: tumor microenvironment
VEGF: vascular endothelial growth factor
VIMP: variable importance

## Supplementary Material

Supplemental Material

## Data Availability

FASTQ files generated by MiSeq were deposited in ArrayExpress under code E-MTAB-12513.

## References

[1] Sung H, Ferlay J, Siegel RL, Laversanne M, Soerjomataram I, Jemal A, Bray F. Global cancer statistics 2020: GLOBOCAN estimates of incidence and mortality worldwide for 36 cancers in 185 countries. CA Cancer J Clin. 2021 May;71(3):209–25. doi:10.3322/caac.21660. 33538338

[2] Siegel RL, Miller KD, Fuchs HE, Jemal A. Cancer statistics, 2021. CA Cancer J Clin. 2021 Jan;71(1):7–33. doi:10.3322/caac.21654. Erratum in: CA Cancer J Clin. 2021 Jul;71(4):359. 33433946

[3] Sohal DPS, Kennedy EB, Cinar P, Conroy T, Copur MS, Crane CH, Garrido-Laguna I, Lau MW, Johnson T, Krishnamurthi S, et al. Metastatic pancreatic cancer: ASCO guideline update. J Clin Oncol. 2020 Aug 5;38(27):3217–3229. doi:10.1200/JCO.20.01364. 32755482 PMC12974607

[4] Chen X, Liu F, Xue Q, Weng X, Xu F. Metastatic pancreatic cancer: mechanisms and detection (review). Oncol Rep. 2021 Nov;46(5):231. doi:10.3892/or.2021.8182. 34498718 PMC8444192

[5] Lucas AL, Kastrinos F. Screening for pancreatic cancer. JAMA. 2019 Aug 6;322(5):407–408. doi:10.1001/jama.2019.9690. 31386115

[6] Yang J, Xu R, Wang C, Qiu J, Ren B, You L. Early screening and diagnosis strategies of pancreatic cancer: a comprehensive review. Cancer Commun (Lond). 2021 Dec;41(12):1257–1274. doi:10.1002/cac2.12204. 34331845 PMC8696234

[7] Redegalli M, Schiavo Lena M, Cangi MG, Smart CE, Mori M, Fiorino C, Arcidiacono PG, Balzano G, Falconi M, Reni M, et al. Proposal for a new pathologic prognostic index after neoadjuvant chemotherapy in Pancreatic Ductal Adenocarcinoma (PINC). Ann Surg Oncol. 2022 June;29(6):3492–3502. doi:10.1245/s10434-022-11413-7. 35230580 PMC9072515

[8] Ren B, Cui M, Yang G, Wang H, Feng M, You L, Zhao Y. Tumor microenvironment participates in metastasis of pancreatic cancer. Mol Cancer. 2018 Jul 30;17(1):108. doi:10.1186/s12943-018-0858-1. 30060755 PMC6065152

[9] Werner J, Combs S, Springfeld C, Hartwig W, Hackert T, Büchler MW. Advanced-stage pancreatic cancer: therapy options. Nat Rev Clin Oncol. 2013;10(6):323–333. doi:10.1038/nrclinonc.2013.66. 23629472

[10] van Zijl F, Krupitza G, Mikulits W. Initial steps of metastasis: cell invasion and endothelial transmigration. Mutat Res. 2011 Jul;728(1–2):23–34. doi:10.1016/j.mrrev.2011.05.002. 21605699 PMC4028085

[11] Fares J, Fares MY, Khachfe HH, Salhab HA, Fares Y. Molecular principles of metastasis: a hallmark of cancer revisited. Signal Transduct Target Ther. 2020 Mar 12;5(1):28. doi:10.1038/s41392-020-0134-x. 32296047 PMC7067809

[12] Liu Q, Zhang H, Jiang X, Qian C, Liu Z, Luo D. Factors involved in cancer metastasis: a better understanding to “seed and soil” hypothesis. Mol Cancer. 2017 Dec 2;16(1):176. doi:10.1186/s12943-017-0742-4. 29197379 PMC5712107

[13] Fu A, Yao B, Dong T, Chen Y, Yao J, Liu Y, Li H, Bai H, Liu X, Zhang Y, et al. Tumor-resident intracellular microbiota promotes metastatic colonization in breast cancer. Cell. 2022;185(8):1356–1372. doi:10.1016/j.cell.2022.02.027. 35395179

[14] He Y, Huang J, Li Q, Xia W, Zhang C, Liu Z, Xiao J, Yi Z, Deng H, Xiao Z, et al. Gut microbiota and tumor immune escape: a new perspective for improving tumor immunotherapy. Cancers. 2022;14(21):5317. doi:10.3390/cancers14215317. 36358736 PMC9656981

[15] Qiu M, Huang K, Liu Y, Yang Y, Tang H, Liu X, Wang C, Chen H, Xiong Y, Zhang J, et al. Modulation of intestinal microbiota by glycyrrhizic acid prevents high-fat diet-enhanced pre-metastatic niche formation and metastasis. Mucosal Immunol. 2019 Jul;12(4):945–957. doi:10.1038/s41385-019-0144-6. 30755716

[16] Cheng P, Wu J, Zong G, Wang F, Deng R, Tao R, Qian C, Chen W, Wang A, Zhao Y, et al. Capsaicin shapes gut microbiota and pre-metastatic niche to facilitate cancer metastasis to liver. Res Sq. 2022;188:106643. 10.1016/j.phrs.2022.106643 36608780

[17] Abdel Sater AH, Bouferraa Y, Amhaz G, Haibe Y, Lakkiss AE, Shamseddine A. From tumor cells to endothelium and gut microbiome: a complex interaction favoring the metastasis cascade. Front Oncol. 2022 May 5;12:804983. doi:10.3389/fonc.2022.804983. 35600385 PMC9117727

[18] Rossi T, Vergara D, Fanini F, Maffia M, Bravaccini S, Pirini F. Microbiota-derived metabolites in tumor progression and metastasis. Int J Mol Sci. 2020 Aug 12;21(16):5786. doi:10.3390/ijms21165786. 32806665 PMC7460823

[19] Sun Y, Wu C, Ma J, Yang Y, Man X, Wu H, Li S. Toll-like receptor 4 promotes angiogenesis in pancreatic cancer via PI3K/AKT signaling. Exp Cell Res. 2016 Oct 1;347(2):274–282. doi:10.1016/j.yexcr.2016.07.009. 27426724

[20] Klindworth A, Pruesse E, Schweer T, Peplies J, Quast C, Horn M, Glöckner FO. Evaluation of general 16S ribosomal RNA gene PCR primers for classical and next-generation sequencing-based diversity studies. Nucleic Acids Res. 2013 Jan 7;41(1):e1. doi:10.1093/nar/gks808. 22933715 PMC3592464

[21] Picchianti-Diamanti A, Panebianco C, Salemi S, Sorgi ML, Di Rosa R, Tropea A, Sgrulletti M, Salerno G, Terracciano F, D’Amelio R, et al. Analysis of gut microbiota in rheumatoid arthritis patients: disease-related dysbiosis and modifications induced by etanercept. Int J Mol Sci. 2018;19(10):2938. doi:10.3390/ijms19102938. 30261687 PMC6213034

[22] Dettling M, Bühlmann P. Finding predictive gene groups from microarray data. J Multivar Anal. 2004;90(1):106–131. doi:10.1016/j.jmva.2004.02.012.

[23] Manchia M, Fontana A, Panebianco C, Paribello P, Arzedi C, Cossu E, Garzilli M, Montis MA, Mura A, Pisanu C, et al. Involvement of gut microbiota in schizophrenia and treatment resistance to antipsychotics. Biomedicines. 2021 Jul 23;9(8):875. doi:10.3390/biomedicines9080875. 34440078 PMC8389684

[24] Fontana A, Manchia M, Panebianco C, Paribello P, Arzedi C, Cossu E, Garzilli M, Montis MA, Mura A, Pisanu C, et al. Exploring the role of gut microbiota in major depressive disorder and in treatment resistance to antidepressants. Biomedicines. 2020 Aug 27;8(9):311. doi:10.3390/biomedicines8090311. 32867257 PMC7554953

[25] Villani A, Fontana A, Barone S, de Stefani S, Primiterra M, Copetti M, Panebianco C, Parri C, Sciannamè N, Quitadamo PA, et al. Identifying predictive bacterial markers from cervical swab microbiota on pregnancy outcome in woman undergoing assisted reproductive technologies. J Clin Med. 2022 Jan 28;11(3):680. doi:10.3390/jcm11030680. 35160131 PMC8836651

[26] Basu S, Kumbier K, Brown JB, Yu B. Iterative random forests to discover predictive and stable high-order interactions. Proc Natl Acad Sci USA. 2018 Feb 20. 115(8):1943–1948. doi:10.1073/pnas.1711236115. 29351989 PMC5828575

[27] De Rosa A, Fontana A, Nuzzo T, Garofalo M, Di Maio A, Punzo D, Copetti M, Bertolino A, Errico F, Rampino A, et al. Machine Learning algorithm unveils glutamatergic alterations in the post-mortem schizophrenia brain. Schizophr (Heidelb). 2022 Feb 25;8(1):8. doi:10.1038/s41537-022-00231-1. PMC8881508 35217646

[28] Zhao L, Yang R, Cheng L, Wang M, Jiang Y, Wang S. LPS-induced epithelial-mesenchymal transition of intrahepatic biliary epithelial cells. J Surg Res. 2011 Dec;171(2):819–825. doi:10.1016/j.jss.2010.04.059. 20691985

[29] Li H, Li Y, Liu D, Liu J. LPS promotes epithelial-mesenchymal transition and activation of TLR4/JNK signaling. Tumour Biol. 2014 Oct;35(10):10429–10435. doi:10.1007/s13277-014-2347-5. 25053598

[30] Pei Z, Lin D, Song X, Li H, Yao H. TLR4 signaling promotes the expression of VEGF and TGFbeta1 in human prostate epithelial PC3 cells induced by lipopolysaccharide. Cell Immunol. 2008;254(1):20–27. doi:10.1016/j.cellimm.2008.06.007. 18649875

[31] Ahn J, Sinha R, Pei Z, Dominianni C, Wu J, Shi J, Goedert JJ, Hayes RB, Yang L. Human gut microbiome and risk for colorectal cancer. J Natl Cancer Inst. 2013 Dec 18;105(24):1907–1911. doi:10.1093/jnci/djt300. 24316595 PMC3866154

[32] Gnanasekaran J, Binder Gallimidi A, Saba E, Pandi K, Eli Berchoer L, Hermano E, Angabo S, Makkawi HA, Khashan A, Daoud A, et al. Intracellular porphyromonas gingivalis promotes the tumorigenic behavior of pancreatic carcinoma cells. Cancers (Basel). 2020 Aug 18;12(8):2331. doi:10.3390/cancers12082331. 32824786 PMC7465784

[33] Keku TO, McCoy AN, Azcarate-Peril AM. *Fusobacterium* spp. And colorectal cancer: cause or consequence? Trends In Microbiology. 2013 Oct;21(10):506–508. doi:10.1016/j.tim.2013.08.004. 24029382 PMC3836616

[34] Udayasuryan B, Ahmad RN, Nguyen TTD, Umaña A, Monét Roberts L, Sobol P, Jones SD, Munson JM, Slade DJ, Verbridge SS. Fusobacterium nucleatum induces proliferation and migration in pancreatic cancer cells through host autocrine and paracrine signaling. Sci Signal. 2022 Oct 18. 15(756):eabn4948. doi:10.1126/scisignal.abn4948. 36256708 PMC9732933

[35] Chen Y, Chen Y, Zhang J, Cao P, Su W, Deng Y, Zhan N, Fu X, Huang Y, Dong W. Fusobacterium nucleatum promotes metastasis in colorectal cancer by activating autophagy signaling via the upregulation of CARD3 expression. Theranostics. 2020 Jan 1;10(1):323–339. doi:10.7150/thno.38870. Erratum in: Theranostics. 2022 Jan 1;12(3):1333-1334. 31903123 PMC6929621

[36] Li Z, Dou L, Zhang Y, He S, Zhao D, Hao C, Song G, Zhang W, Liu Y, Wang G. Characterization of the oral and esophageal microbiota in esophageal precancerous lesions and squamous cell carcinoma. Front Cell Infect Microbiol. 2021 Sep 15;11:714162. doi:10.3389/fcimb.2021.714162. 34604107 PMC8479167

[37] Zhang Q, Wu Y, Wang J, Wu G, Long W, Xue Z, Wang L, Zhang X, Pang X, Zhao Y, et al. Accelerated dysbiosis of gut microbiota during aggravation of DSS-induced colitis by a butyrate-producing bacterium. Sci Rep. 2016 June 6;6(1):27572. doi:10.1038/srep27572. 27264309 PMC4893749

[38] Nie K, Ma K, Luo W, Shen Z, Yang Z, Xiao M, Tong T, Yang Y, Wang X. Roseburia intestinalis: a beneficial gut organism from the discoveries in genus and species. Front Cell Infect Microbiol. 2021 Nov 22;11:757718. doi:10.3389/fcimb.2021.757718. 34881193 PMC8647967

[39] Farrow B, Rychahou P, O’Connor KL, Evers BM. Butyrate inhibits pancreatic cancer invasion. J Gastrointestinal Surg. 2003 Nov;7(7):864–870. doi:10.1007/s11605-003-0031-y. 14592659

[40] Liu X, Cheng YW, Shao L, Sun SH, Wu J, Song QH, Zou HS, Ling ZX. Gut microbiota dysbiosis in Chinese children with type 1 diabetes mellitus: an observational study. World J Gastroenterol. 2021 May 21;27(19):2394–2414. doi:10.3748/wjg.v27.i19.2394.Erratum in: World J Gastroenterol. 2022 Jul 7;28(25):3006-3007. 34040330 PMC8130045

[41] Li Q, Li Y, Wang Y, Xu L, Guo Y, Wang Y, Wang L, Guo C. Oral administration of Bifidobacterium breve promotes antitumor efficacy via dendritic cells-derived interleukin 12. Oncoimmunology. 2021 Jan 15;10(1):1868122. doi:10.1080/2162402X.2020.1868122. 33537172 PMC7833736

[42] Zhao H, Li D, Liu J, Zhou X, Han J, Wang L, Fan Z, Feng L, Zuo J, Wang Y. Bifidobacterium breve predicts the efficacy of anti-PD-1 immunotherapy combined with chemotherapy in Chinese NSCLC patients. Cancer Medicine. 2022 Oct 7;12(5):6325–6336. doi:10.1002/cam4.5312. 36205311 PMC10028067

[43] Xu Y, Jia Y, Chen L, Gao J, Yang D. Effect of Streptococcus anginosus on biological response of tongue squamous cell carcinoma cells. BMC Oral Health. 2021 Mar 20;21(1):141. doi:10.1186/s12903-021-01505-3. 33743656 PMC7981962

[44] Huang B, Huang H, Zhang S, Zhang D, Shi Q, Liu J, Guo J. Artificial intelligence in pancreatic cancer. Theranostics. 2022 Oct 3;12(16):6931–6954. doi:10.7150/thno.77949. 36276650 PMC9576619

[45] Herner A, Sauliunaite D, Michalski CW, Erkan M, De Oliveira T, Abiatari I, Kong B, Esposito I, Friess H, Kleeff J. Glutamate increases pancreatic cancer cell invasion and migration via AMPA receptor activation and Kras-MAPK signaling. Int J Cancer. 2011 Nov 15;129(10):2349–2359. doi:10.1002/ijc.25898. 21207374

[46] Cooke M, Kazanietz MG. Overarching roles of diacylglycerol signaling in cancer development and antitumor immunity. Sci Signal. 2022 Apr 12;15(729):eabo0264. doi:10.1126/scisignal.abo0264. 35412850 PMC12985357

[47] Zhao Y, Sun H, Li X, Liu Q, Liu Y, Hou Y, Jin W. DGKZ promotes TGFβ signaling pathway and metastasis in triple-negative breast cancer by suppressing lipid raft-dependent endocytosis of TGFβR2. Cell Death Dis. 2022;13(2):105. doi:10.1038/s41419-022-04537-x. 35115500 PMC8814002

[48] Tian X, Wu L, Jiang M, Zhang Z, Wu R, Miao J, Liu C, Gao S. Corrigendum: downregulation of GLYAT facilitates tumor growth and metastasis and poor clinical outcomes through the PI3K/AKT/Snail pathway in human breast cancer. Front Oncol. 2022 Apr 6;12:793448. doi:10.3389/fonc.2022.793448. 35463385 PMC9020255

[49] Luo X, Cheng C, Tan Z, Li N, Tang M, Yang L, Cao Y. Emerging roles of lipid metabolism in cancer metastasis. Mol Cancer. 2017 Apr 11;16(1):76. doi:10.1186/s12943-017-0646-3. 28399876 PMC5387196

[50] Notarnicola M, Lorusso D, Tutino V, De Nunzio V, De Leonardis G, Marangelli G, Guerra V, Veronese N, Caruso MG, Giannelli G. Differential tissue fatty acids profiling between colorectal cancer patients with and without synchronous metastasis. Int J Mol Sci. 2018 Mar 23;19(4):962. doi:10.3390/ijms19040962. 29570667 PMC5979339

[51] Galluzzi L, Vacchelli E, Michels J, Garcia P, Kepp O, Senovilla L, Vitale I, Kroemer G. Effects of vitamin B6 metabolism on oncogenesis, tumor progression and therapeutic responses. Oncogene. 2013 Oct 17;32(42):4995–5004. doi:10.1038/onc.2012.623. 23334322

[52] D’Eliseo D, Velotti F. Omega-3 fatty acids and cancer cell cytotoxicity: implications for multi-targeted cancer therapy. J Clin Med. 2016 Jan 26;5(2):15. doi:10.3390/jcm5020015. 26821053 PMC4773771

[53] Jóźwiak M, Filipowska A, Fiorino F, Struga M. Anticancer activities of fatty acids and their heterocyclic derivatives. Eur J Pharmacol. 2020 Mar 15;871:172937. doi:10.1016/j.ejphar.2020.172937. 31958454

