# Supplementary material for: A powerful machine learning approach to identify interactions of differentially abundant gut microbial subsets in patients with metastatic and non-metastatic pancreatic cancer
Source: Gut Microbes. 2024 Jul 7;16(1):2375483. doi: 10.1080/19490976.2024.2375483 (PMC11229760; doi:10.1080/19490976.2024.2375483)
Supplement: Supplemental Material [file KGMI_A_2375483_SM3378.zip › Supplementary Methods.docx]

**Supplementary Methods**

**1 Supplemental Statistical Methods**

*1.1 The Random Forest algorithm: an overview*

Random Forest (RF) is a popular machine learning algorithm that consists of an ensemble of decision trees. In RFs, it is not imperative to perform cross-validation or a separate test set to obtain an unbiased estimate of the test set error, which is estimated internally during the execution, because each tree is built on a bootstrap sample (in which observations are extracted randomly with replacement) from the original dataset. Approximately one-third of the observations are left out of the bootstrap sample and are not used in the construction of each tree; this is the so-called out of bag (OOB) data. The tree-growing algorithm recursively splits the bootstrap sample data into subgroups, choosing the best binary split for each variable considered, to identify the most homogeneous sets within each tree node and the most heterogeneous sets between nodes (Gini Impurity measure). To split each tree node, if there are M candidate input variables in the dataset, only m<<M variables are randomly selected (each time, among the M) to be considered for that split. The tree was grown until the minimum node size was reached (user-defined stop criterion). The OOB data of each tree was used to obtain an unbiased estimate of the RF prediction error (i.e., the Brier Score) as trees were added to the forest; each tree provides the probability of having metastases for each observation in the OOB data. The final individual probability (of having metastases) is the average of all probabilities over all trees in the forest, estimated for that observation. The Brier Score is the mean square difference between individual predicted probabilities and individual actual results and varies from 0 (i.e., RF is perfectly calibrated) to 1 (i.e. RF is perfectly miscalibrated). The OOB data were also used to obtain a variable importance (VIMP) estimate, which is defined as the average over all trees of the difference in prediction error before and after the permutation of the values of each variable in the OOB data within each tree.

*1.2 Technical details of the iterative Random Forest algorithm*

Iterative RF (iRF) is a generalization of the Random Forest (RF) algorithm and is commonly used to train a set of feature-weighted decision trees to detect stable and high-order interactions [1]. In the iRF algorithm, an RF is run iteratively K times. In the first iteration, a subsample of candidate variables (i.e. features) is randomly selected at each split of a decision tree. Based on the variable importance values and a regularization factor, which may penalize the variable selection at each split of each decision tree, new (updated) weights are assigned (at each iteration) to all candidate variables such that those with higher weights are more likely to be selected than the others. As a result, the weights of most non-predictive variables are gradually set to zero during the iterations. Therefore, in the last iteration, the iRF includes regularized trees, and the decision rules extracted from such feature-weighted RF are mapped [2]. This mapping allows the identification of prevalent interactions in the RF through a computationally efficient algorithm (i.e., the generalized random intersection Trees – RIT – algorithm [1]) that searches for high-order interactions in binary data. A bagging step assesses the stability of the recovered interactions with respect to the bootstrap perturbation of the data. The proportion of times (out of B bootstrap samples) an interaction appears as an output of the RIT defines a “stability score” (i.e., 0=totally unstable interaction, 1=totally stable interaction).

*1.3 Tuning the Iterative Random Forest algorithm*

To enable the iRF training, the following parameters have to be set, some of which have to be fixed in advance, while others can be set at the end of a “tuning phase”: 1) the choice of the number of iterations (parameter K) of the iRF: from 1 to 100 iterations were evaluated and the choice of the optimal number of iterations is determined after the tuning phase; 2) the choice of the number of the trees included in the random forest (within each iteration): this parameter was set at 100’000 trees (fixed in advance); 3) the criteria used to select the number of features that possibly split at in each node of the tree included into the forest: the square root of the number of variables is considered (fixed in advance); 4) the choice of the number of outer-layer bootstrap samples: this parameter was set at 30 samples (fixed in advance); 5) the choice of the splitting criterion: Gini impurity measure (fixed in advance); 6) the choice of minimum node size: at least 5 observation must be included the final leaves of each tree in the forest (fixed in advance); 7) the choice of the value of the regularization factor: values 1.0 (no regularization), 0.9 (weak regularization) to 0.8 (moderate regularization) were evaluated and the choice of the optimal regularization factor value is determined after the tuning phase; 8) the choice of whether the RF should return a binary classification or an individual probability estimate (of having metastases) as output: probability forests were grown along with the estimation of Brier Score (fixed in advance); 9) the choice of the metric used to compute the variable importance measure: the permutation variable importance (i.e. permuting the OOB cases) was fixed in advance. Therefore, the “tuning phase” finally consists a grid search for the optimal parameter combination (i.e., the number of iterations and the value of the regularization factor) that minimizes the Brier Score achieved by iRF in the OOB data (see Supplemental Figure 1). The following table summarizes all nine parameters (six are required and three are optional) to be provided to the iRF, together with their possible values:

|  | **Parameters (description)** | **Setting values** |
| --- | --- | --- |
| **Required** | The number of random forest iterations | Tuning phase: from 1 to 100 iterations were evaluated |
|  | The number of the trees included into the forest | Fixed value: 100’000 trees at each iteration |
|  | The number of (randomly chosen) features that possibly split at in each node of the tree | Fixed value: at the first iteration, when all variables have the same probability of being selected by the algorithm, the square root of the number of variables is considered (default option). After that, the number of candidate variables that are randomly chosen for each tree is dramatically reduced as the weights assigned to each iteration are updated |
|  | The number of outer-layer bootstrap samples | Fixed value: 30 samples |
|  | The splitting criterion | Fixed value: each node in the tree was split by the feature that minimize the Gini impurity measure |
|  | Minimum node size | Fixed value: the final leaves of each tree in the forest must include at least 5 observations |
| **Optional** | Variable regularization factor: regularization works by penalizing new features by multiplying the splitting criterion by a factor, in order to perform an efficient feature selection, so that "regularized trees" are built. This procedure can enhance the weight update phase that occurs at each iteration. The key idea is to penalize selecting a new feature for splitting when its gain (e.g. information gain) is similar to the features used in previous splits | Tuning phase: three possible values were evaluated: 1.0 (no regularization), 0.9 (weak regularization), 0.8 (moderate regularization) |
|  | Should the forest return a binary classification or an individual probability? In the probability forest, each tree of the forest returns a probability estimate (of having metastases) and these estimates are averaged for the forest probability estimate | Fixed value: probability forests were grown along with the estimation of Brier Score (i.e. prediction error) |
|  | Variable Importance measure | Fixed value: permutation variable importance (i.e. permuting the OOB cases) |

**2 Supplemental Figures**

**Supplemental Figure 1**. Traceplots of the out of bag Brier score (i.e., prediction error) against an increasing number of iterative Random Forest iterations and three values of the regularization factor (the lower the value, the stronger the regularization) for Phylum (**A**), Family (**B**), Genus (**C**), and Species (**D**). The arrow identifies the optimal combination of both parameters that minimizes the Brier score


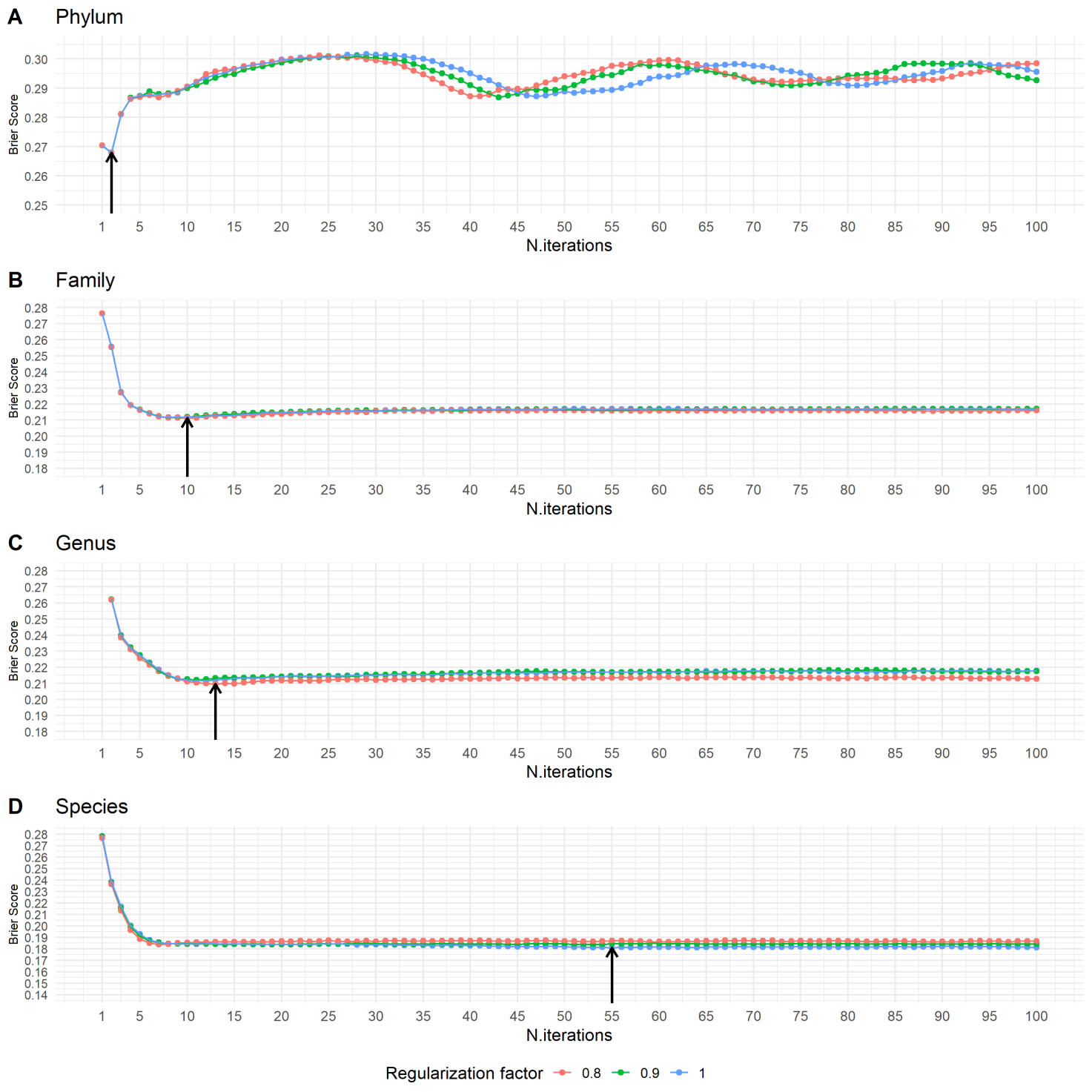


**Figure legend**: At phylum level, the minimum Brier Score was reached at 2 iterations and at a regularization factor of 0.9 (weak regularization) (**A**). At Family level, the minimum Brier Score was reached at 10 iterations and at a regularization factor of 0.9 (weak regularization) (**B**). At Genus level, the minimum Brier Score was reached at 13 iterations and at a regularization factor of 0.8 (moderate regularization) (**C**). At Species level, the minimum Brier Score was reached at 55 iterations and at a regularization factor of 1 (no regularization) (**D**).

3 Supplemental Metabolomics Methods

300 µl Isopropanol were added to 100 mg of feces along with Internal Standards (IS). 1 µg of each IS (Tryptophan(indole)-d5; Phenylalanine-15N; Stearic Acid-d3; Phosphoetanolamine-d4) were added to each sample with 20 µl of EquiSPLASH (Avanti Polar). Samples were vortexed for 2 min to obtain a homogeneous mixture, sonicated on ice for 5 min, then centrifuged at 14000g for 15 min at 4 °C. The supernatants were filtered with 0.45 µm filter. After filtration the samples were diluted 1:1 with Isopropanol. The metabolomic profile of feces from 10 patients with pancreatic tumor (PC) and 10 with metastatic pancreatic tumor (MET) was analyzed by ultra-high pressure liquid chromatography (UPLC 1290 system, Agilent Technologies) directly connected to mass spectrometry (TripleTOF 5600+ mass spectrometer, SCIEX equipped with an electrospray ionization source (ESI)). Quality control (QC) samples were prepared as pool of all the samples, by mixing equal volume of each extracted sample and analyzed within the queue. Chromatographic separations occurred on an Acquity BEH AMIDE (100 x 2.1 mm, 1.7 µm, Waters) capillary column. A gradient of solvent A (acetonitrile containing 0.1% formic acid) and B (water containing 0.1% formic acid) was used to achieve separation (600 µl/min as flow rate): 1 minute at 2% B, from 2% B to 60% B in 10 minutes, 2 minutes hold at 60% B, in 0.50 minutes to 2% B, 3 minutes hold at 2% B. The column temperature was set at 40°C, while the autosampler was set at 4°C. 8 µl of samples were injected. Full scan spectra were acquired in the mass range from *m*/*z* 50 to 500 with a SWATH modality acquisition of 10 windows. The source parameters were: Gas 1: 33 psi, Gas 2: 58 psi, Curtain gas: 35 psi, Temperature: 500 °C and ISVF (IonSpray Voltage Floating): 5500 V (-4500 V for negative polarity), DP: 80 V, CE: 35 V with a spread of 15V. Lipidomic profiling was obtained by using Acquity BEH C8 (100 x 2.1 mm, 1.7 µm, Waters) capillary column. A gradient of solvent A (water containing 0.1% formic acid) and B (methanol/isopropanol = 85/15 containing 0.1% formic acid) was used to achieve separation (400 µl/min as flow rate): 1 minute at 75% B, from 75% B to 85% B in 2 minutes, in 12 minutes to 99.9% B, 1 minute hold at 99.9% B, in 1 minute to 75% B, 2 minutes hold at 75% B. The column temperature was set at 50°C, while the autosampler was set at 4°C. 10 µl of samples were injected. Full scan spectra were acquired in the mass range from *m*/*z* 150 to 1200 with a SWATH modality acquisition of 24 windows. The source parameters were: Gas 1: 33 psi, Gas 2: 58 psi, Curtain gas: 35 psi, Temperature: 500 °C and ISVF (IonSpray Voltage Floating): 4500 V (-4500 V for negative polarity), DP: 80 V, CE: 45 V with a spread of 15V. The .wiff files acquired on the mass spectrometer were converted to .abf files using Reifycs Analysis Base File Converter and analysed with MS-DIAL v. 4.9 [3] for peak picking, gap filling, alignment. The identification was performed using MS/MS Positive Public library VS17, MS/MS Negative Public library VS17 and Tandem Mass Spectra Atlas-VS69 in positive and negative modalities. The MS-DIAL output was manually inspected to verify the annotation of the metabolites. The annotated metabolites were then statistically analysed by our in-house developed R pipeline: heatmaps were generated upon t-test among the two experimental sample groups (p<0.05).

**References for Supplemental Methods**

[1] Basu S, Kumbier K, Brown JB, Yu B. Iterative random forests to discover predictive and stable high-order interactions. Proc Natl Acad Sci U S A. 2018 Feb 20;115(8):1943-1948.

[2] Deng, H. T. & Runger, G. Feature Selection via Regularized Trees. The 2012 International Joint Conference on Neural Networks (IJCNN). 2012, pp. 1-8, doi: 10.1109/IJCNN.2012.6252640

[3] Tsugawa H, Cajka T, Kind T, Ma Y, Higgins B, Ikeda K, Kanazawa M, VanderGheynst J, Fiehn O, Arita M. MS-DIAL: data-independent MS/MS deconvolution for comprehensive metabolome analysis. Nat Methods. 2015 Jun;12(6):523-6.
